# A haplotype reference panel constructed from 490,319 UK Biobank genomes improves genotype imputation for global populations

**DOI:** 10.1038/s41588-026-02819-0

**Published:** 2026-10-30

**Authors:** Lars Wienbrandt, Christoph Prieß, Marcus Schmöhl, Volker Neff, Maria Gretsova, Guillermo Torres, Eike Matthias Wacker, Andre Franke, David Ellinghaus

**Affiliations:** Institute of Clinical Molecular Biology, Kiel University and University Medical Center Schleswig-Holstein, Kiel, Germany

**Keywords:** Genome-wide association studies, Software, Genetics research, Epidemiology

## Abstract

Here we constructed a genotype imputation reference panel from whole-genome sequencing data from 490,319 UK Biobank (UKB) participants^1^ and enabled both standalone and cloud-based phasing and imputation through EagleImp^2^ and EagleImp-RAP within the UK Biobank Research Analysis Platform. The UKB reference panel achieves lower phasing switch error rates than the widely used TOPMed r3 panel (133,597 individuals) for all global superpopulations except AMR and for 25 of 26 1000 Genomes Project subpopulations. Imputation accuracy, measured by MAE, improved for 4 of 5 superpopulations and 12 subpopulations for variants with minor allele frequencies down to 0.01%. Estimated squared correlation further improved across all 5 superpopulations and 22 subpopulations, with the largest gains in East and South Asian populations. Reimputation of COVID-19 genome-wide association study datasets from Italy, Germany, Spain and Norway^3,4^ demonstrated that common-variant imputation remains unsaturated, enhancing the discovery of genome-wide significant loci and improving fine-mapping resolution.

## Abstract (web summary)

A haplotype reference panel constructed from whole-genome sequencing data from 490,319 UK Biobank participants improves genotype imputation accuracy across diverse populations and achieves lower phasing switch error rates than existing approaches.

## Main

Imputation services have been developed that allow researchers to easily conduct genome-wide phasing and imputation using reference panels from different genome sequencing projects (for example, the Haplotype Reference Consortium^5^ and the Trans-Omics for Precision Medicine (TOPMed) program^6^) without using their own computational resources (Supplementary Notes 1 and 2, Supplementary Figs. 1–4 and Supplementary Table 1). However, genome-wide sequencing data from the TOPMed r3 panel (133,597 genomes; the largest imputation panel so far) are only partially available through the database of Genotypes and Phenotypes, and the panel was assembled from more than 80 individual studies (the so-called TOPMed projects), which prevents its use on standalone servers or cloud-based research analysis platforms such as the UK Biobank Research Analysis Platform (UKB-RAP).

**Extended Data Fig. 1:**
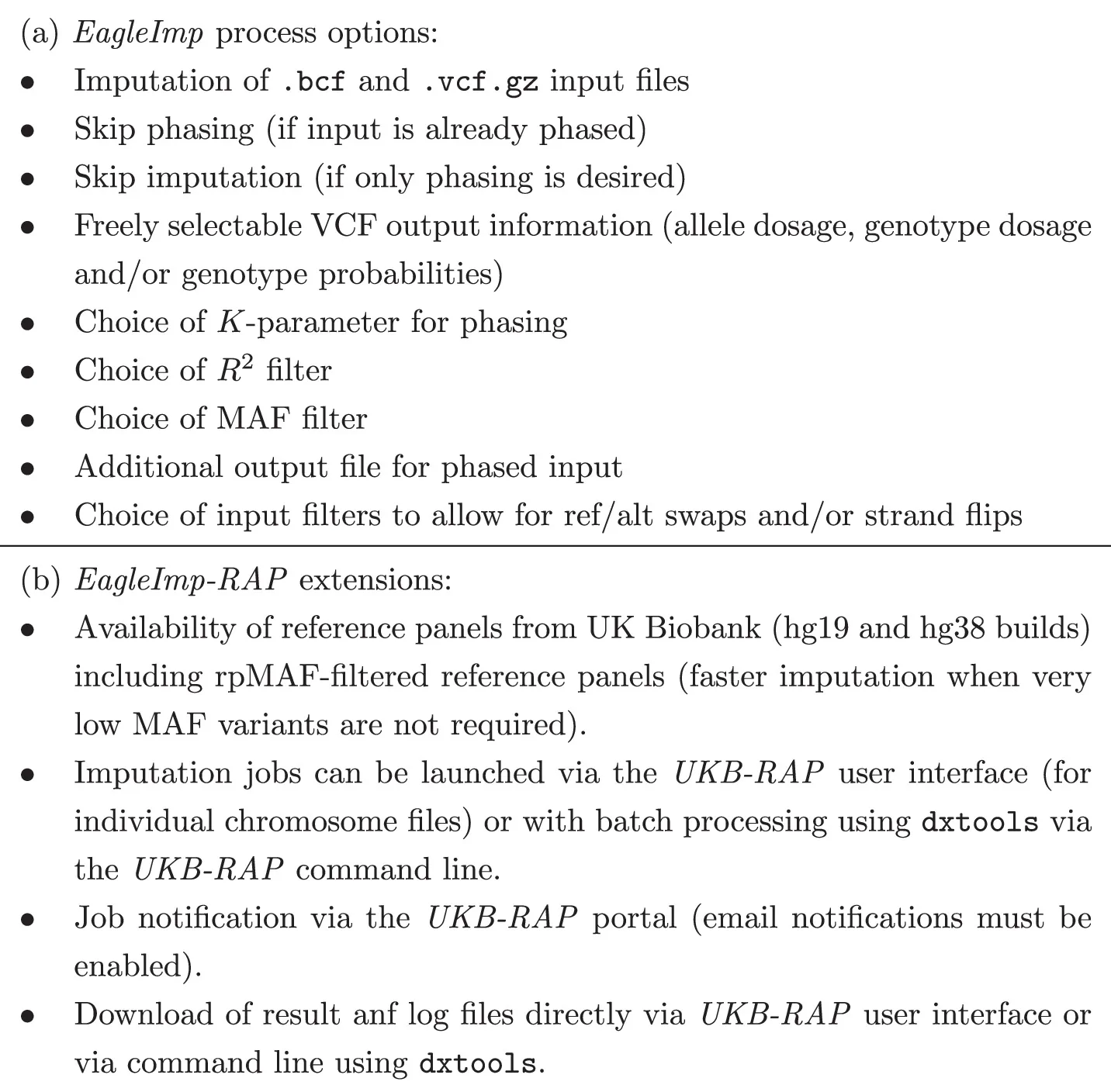
New functionalities added to the EagleImp software framework to enable efficient reference-based phasing and imputation with the UK Biobank reference panels. **a**, List of EagleImp process options. **b**, Summary of additional execution capabilities developed for running EagleImp within the UK Biobank Research Analysis Platform (EagleImp-RAP).

In this study, we constructed whole-genome haplotype reference panels from 490,319 UKB genomes comprising more than 361 million genetic variants^1^—to our knowledge, the largest imputation panels generated to date (Methods, Table 1, Supplementary Note 3 and Supplementary Table 2). To enable reference-based phasing and imputation with these panels, we extended the EagleImp software framework^2^ with new process options and with cloud-execution capabilities for running EagleImp within the UKB-RAP (Extended Data Fig. 1). These new functionalities operate both on the project’s secure standalone compute server—used here for all UKB-based benchmark analyses—and in an implementation of EagleImp inside the UKB-RAP (EagleImp-RAP), enabling reproducibility directly within the UKB-RAP (Methods). Our goal was to provide the UKB research community with a highly accurate imputation solution for diverse global populations, while enabling the use of the enhanced phasing and imputation features implemented in EagleImp.

**Table 1:** Summary of the UKB imputation reference panels constructed in this study

Name	Description	Samples	Variants
UKB complete	Complete UKB reference panel, hg38	490,319	361,441,468
	Complete UKB reference panel, hg19	490,319	359,383,785
UKB MAF0.0001	UKB reference panel filtered for rpMAF ≥ 0.01%, hg38	490,319	51,939,464
	UKB reference panel filtered for rpMAF ≥ 0.01%, hg19	490,319	51,632,019

Reference panels were generated from UKB GraphTyper genotype-level variant data for chromosomes 1–22 and chromosome X (490,319 individuals; Methods). Four panel versions were created, differing only in their rpMAF thresholds and genome builds (hg19 and hg38). The two hg38 panels formed the basis of all phasing and imputation analyses presented in this study.

**Extended Data Fig. 2:**
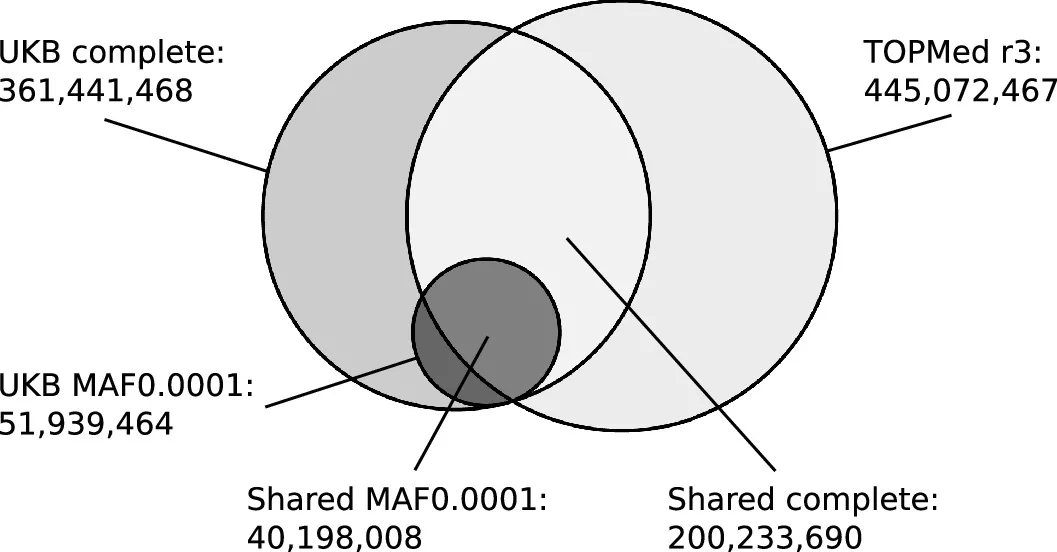
Venn diagram comparing the number of variants in the UK Biobank reference panels (hg38 build) and the TOPMed r3 reference panel. UKB complete shares 200,233,690 variants with TOPMed, while UKB MAF0.0001 shares 40,198,008 variants.

For our first benchmark of imputation quality, we extracted commonly used input variants for imputation available on the Illumina Global Screening Array^7^ from the 1000 Genomes reference panel data and lifted them to genome build GRCh38 using the UCSC liftover tool^8^. We performed imputations separately for the 5 superpopulation datasets (African (AFR), American (AMR), East Asian (EAS), European (EUR) and SAS) and for the 26 subpopulation datasets (Supplementary Table 3), first, with EagleImp and our complete UKB reference panel, second, with EagleImp and our reference panel minor allele frequency (rpMAF)-filtered UKB reference panel and, third, with the TOPMed Imputation Server that uses the TOPMed r3 reference panel. From each imputation run, we calculated mean imputation-estimated *R*^2^ values from reported per-variant *R*^2^ values for three rpMAF categories starting from 0.01%. rpMAF categories are based on the UKB reference panel as per-variant rpMAFs from the TOPMed r3 panel are not publicly available. For each rpMAF category, we calculated the percentage change in imputation quality of EagleImp relative to the TOPMed Imputation Server based on variants shared across the panels (Extended Data Fig. 2). Please refer to Supplementary Note 4 for details on the conducted benchmarks.

**Extended Data Fig. 3:**
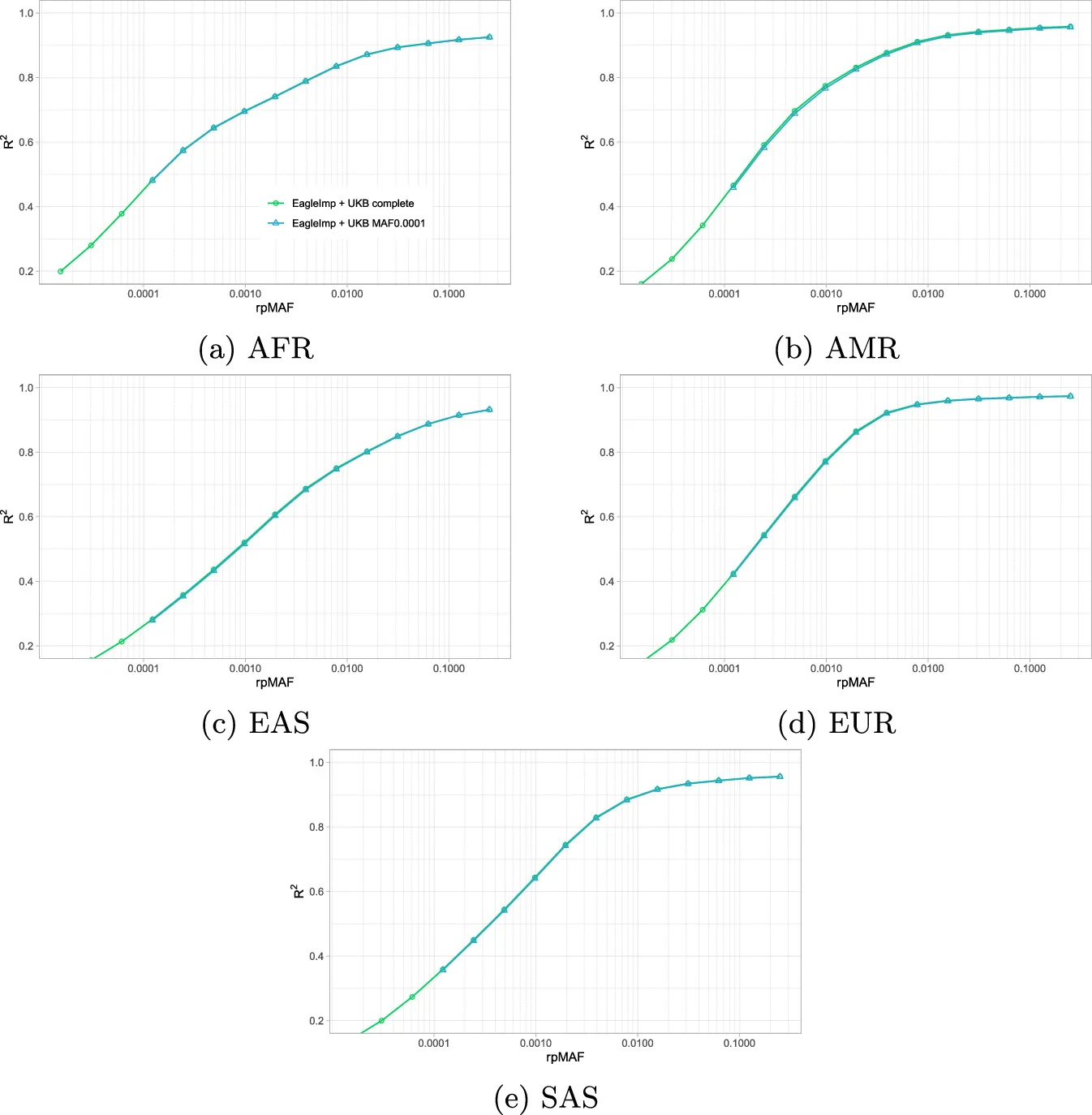
Estimated *R*^2^ distribution for EagleImp + UKB complete panel and EagleImp + UKB MAF0.0001 panel for the benchmark datasets from the five superpopulations of the 1000 Genomes Project data. **a**–**e**, The results demonstrate no significant difference in mean *R*^2^ values for both reference panels. Note that for the UKB MAF0.0001 panel, variants with an rpMAF < 0.0001 are not imputed. Source data

Comparing the first two EagleImp imputation results showed that using the rpMAF-filtered UKB reference panel does not lead to a considerable reduction in estimated imputation *R*^2^ (Extended Data Fig. 3 and Supplementary Table 4). Thus, users who only wish to impute variants with rpMAF ≥ 0.01% (which typically achieve high estimated *R*^2^) may use the rpMAF-filtered UKB panel for faster imputation without noticeable loss of accuracy.

**Fig. 1:**
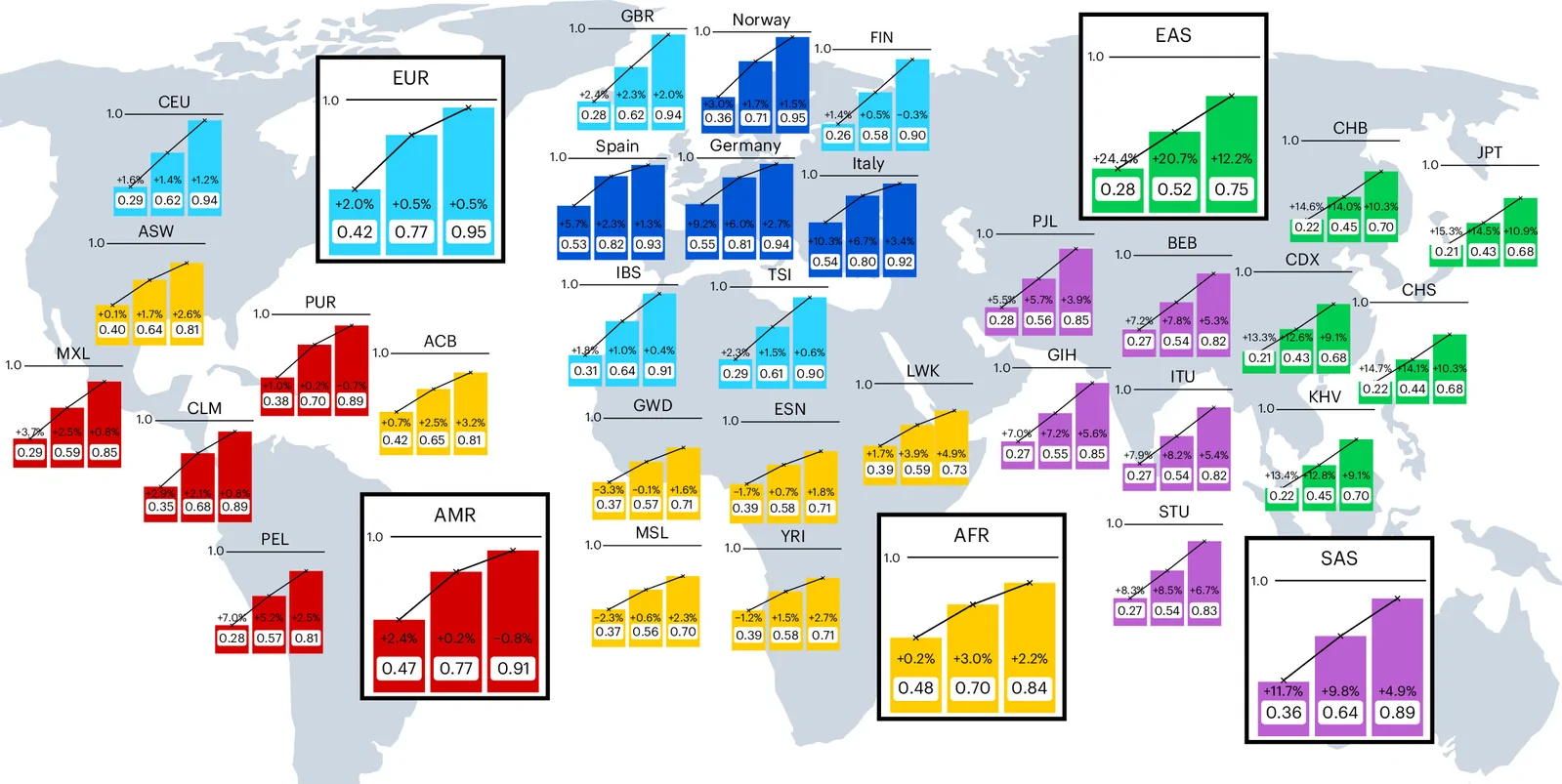
Global comparison of imputation accuracy between EagleImp and TOPMed. World map illustrating the mean estimated imputation R2 reported from EagleImp and the improvement of EagleImp with the complete UKB reference panel (UKB complete) compared to the mean estimated R2 reported from the TOPMed Imputation Server for 35 benchmark datasets from around the world (Supplementary Table 3). Each (super)population shows three bars, where the first (left) bar indicates the mean *R*^2^ for variants with an rpMAF ≥ 0.01%, the second (middle) bar indicates the mean *R*^2^ for rpMAF ≥ 0.1% and the third (right) bar indicates the mean *R*^2^ for rpMAF ≥ 1% computed across all imputed variants. Percentages indicate the improvement of EagleImp in reported estimated *R*^2^ in relation to the TOPMed Imputation Server for imputed variants shared between the UKB reference panel and the TOPMed reference panel (Extended Data Fig. 2). Genome-wide SNP data from (super)populations are taken from the 1000 Genomes Project reference data reduced to Illumina GSA-chip variants. The datasets from Germany, Italy, Norway and Spain are GSA-typed GWAS case-control datasets (see Supplementary Note 4 for details). GSA, Global Screening Array. Source data

**Extended Data Fig. 4:**
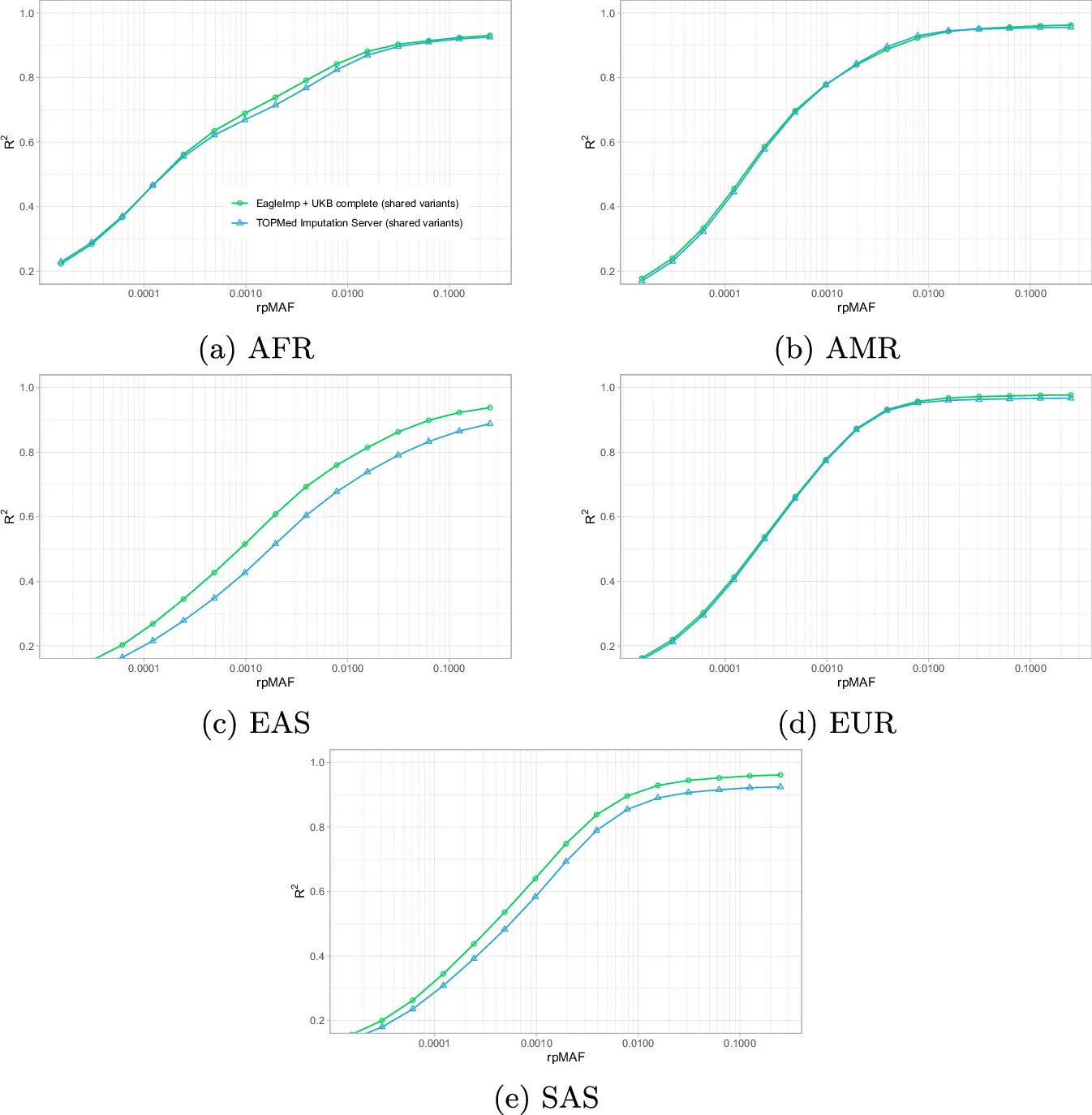
Estimated *R*^2^ distribution for EagleImp + UKB complete panel in comparison to TOPMed for the benchmark datasets from the five superpopulations of the 1000 Genomes Project data. **a**–**e**, Only variants shared between the UKB panel and TOPMed were considered. Reference panel MAF (rpMAF) was taken from the UKB panel. Source data

Comparing the estimated *R*^2^ distributions from EagleImp (complete UKB panel) with those from the TOPMed Imputation Server revealed higher imputation quality for all five superpopulations across the entire allele frequency spectrum, except for a decrease for AMR (−0.76%) for rpMAF ≥ 1%. The largest gain was observed for East Asian (EAS) samples (+24.39% for rpMAF ≥ 0.01%). For rpMAF ≥ 0.01%, imputation quality improved in 22 of 26 subpopulations (max = +15.25% for JPT; min = +0.10% for ASW) and decreased in 4 (worst = −3.33% for GWD). For rpMAF categories of ≥0.1% and ≥1%, 25 and 24 of 26 populations, respectively, showed improved imputation quality. See Fig. 1, Extended Data Fig. 4 and Supplementary Table 5 for details.

Estimated *R*^2^ is the key postimputation quality metric because it is available for each variant from the reference panel and is routinely used to exclude poorly imputed variants in genome-wide association study (GWAS) analyses. The average estimated *R*^2^ for a given MAF category provides a summary of overall imputation quality within that category, whereas estimated *R*^2^ serves as a variant-wise quality criterion for postimputation filtering (Extended Data Fig. 1, EagleImp features). When ground-truth haplotypes with accurate phase information are available (the 1000 Genomes Project haplotypes incorporate trio-based phasing information and are available across diverse populations), direct measures such as switch error rate (SER), mean absolute error (MAE) and correlation *r*^2^ can additionally quantify phasing and imputation performance (Supplementary Note 4).

**Extended Data Fig. 5:**
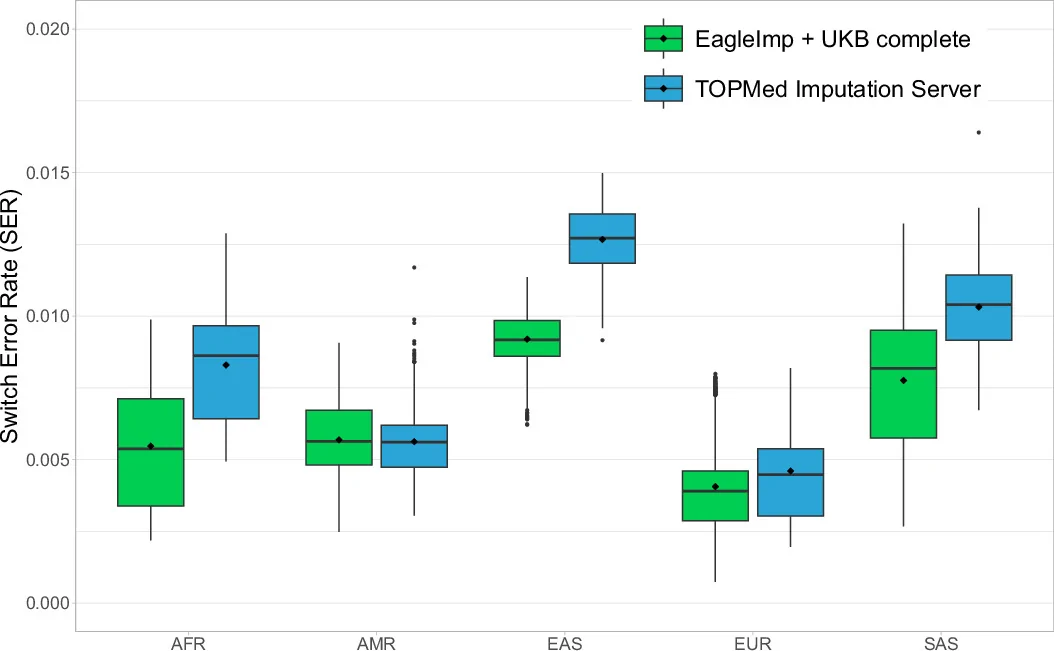
Box plots of the switch error rate (SER) for the five superpopulation benchmark datasets from the 1000 Genomes Project imputed with EagleImp and the complete UK Biobank reference panel (UKB complete) in comparison to TOPMed. SER is measured to the 1000 Genomes reference (ground truth) and is based on all typed (input) variants shared with the TOPMed r3 reference and the 1000 Genomes reference. The number of shared variants is *n* = 540,537. The box shows the inner quartiles, and the whiskers indicate 1.5× the interquartile range. The bar indicates the median, and the mean is represented by the black diamond. Source data

**Extended Data Fig. 6:**
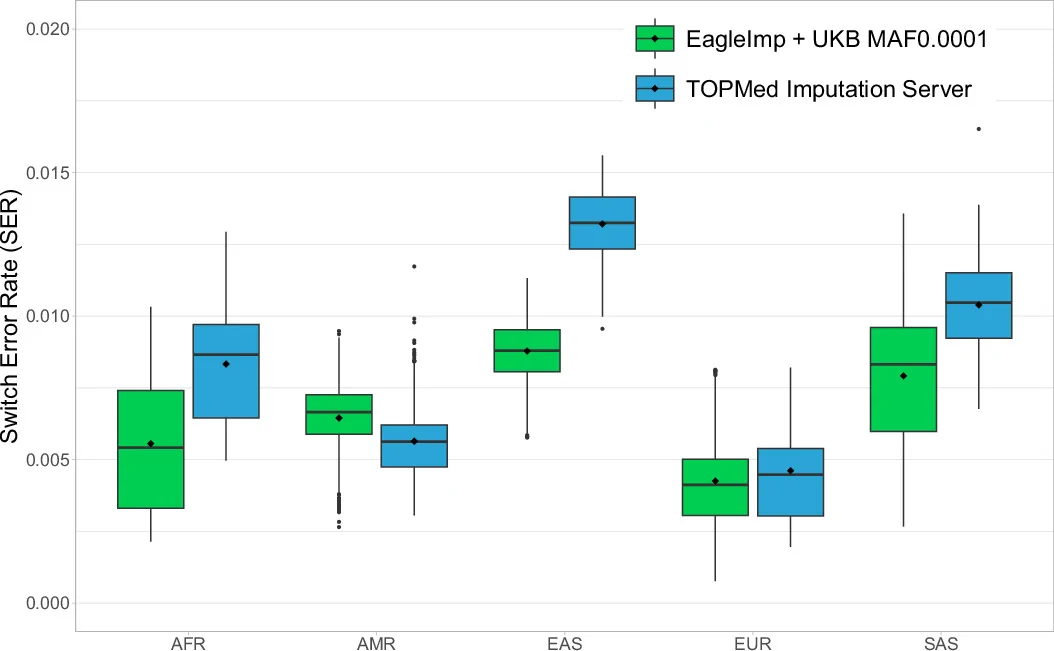
Box plots of the switch error rate (SER) for the five superpopulation benchmark datasets from the 1000 Genomes Project imputed with EagleImp and the rpMAF-filtered UK Biobank reference panel (UKB MAF0.0001) in comparison to TOPMed. SER is measured to the 1000 Genomes reference (ground truth) and is based on all typed (input) variants shared with the TOPMed r3 reference and the 1000 Genomes reference. The number of shared variants is *n* = 540,537. The box shows the inner quartiles, and the whiskers indicate 1.5× the interquartile range. The bar indicates the median, and the mean is represented by the black diamond. Source data

**Extended Data Fig. 7:**
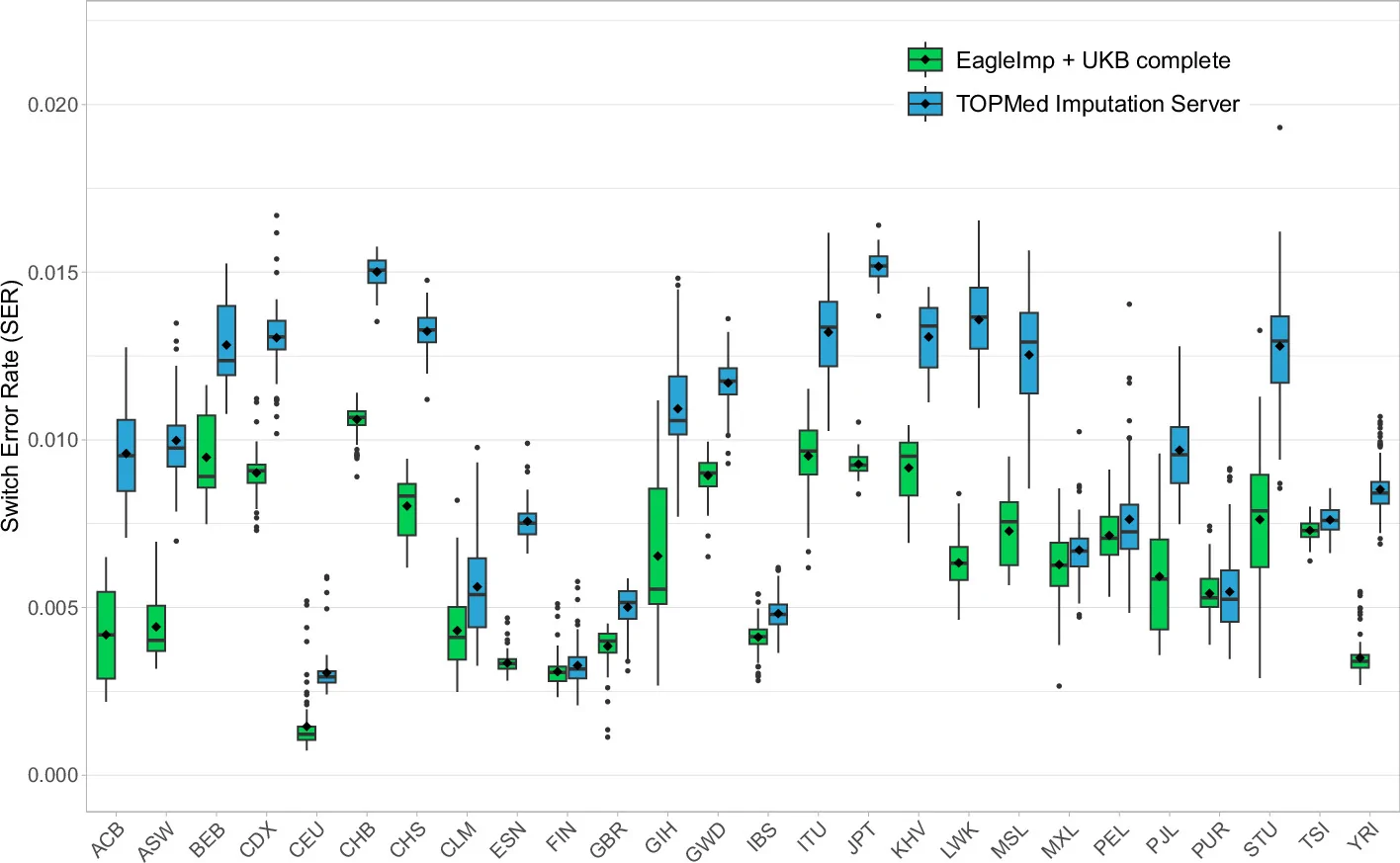
Box plots of the switch error rate (SER) for the 26 subpopulation benchmark datasets from the 1000 Genomes Project imputed with EagleImp and the complete UK Biobank reference panel (UKB complete) in comparison to TOPMed. SER is measured against the 1000 Genomes reference (ground truth) and is based on all typed (input) variants shared with the TOPMed r3 reference and the 1000 Genomes reference. The number of shared variants is *n* = 540,537. The box shows the inner quartiles, and the whiskers indicate 1.5× the interquartile range. The bar indicates the median, and the mean is represented by the black diamond. Source data

**Extended Data Fig. 8:**
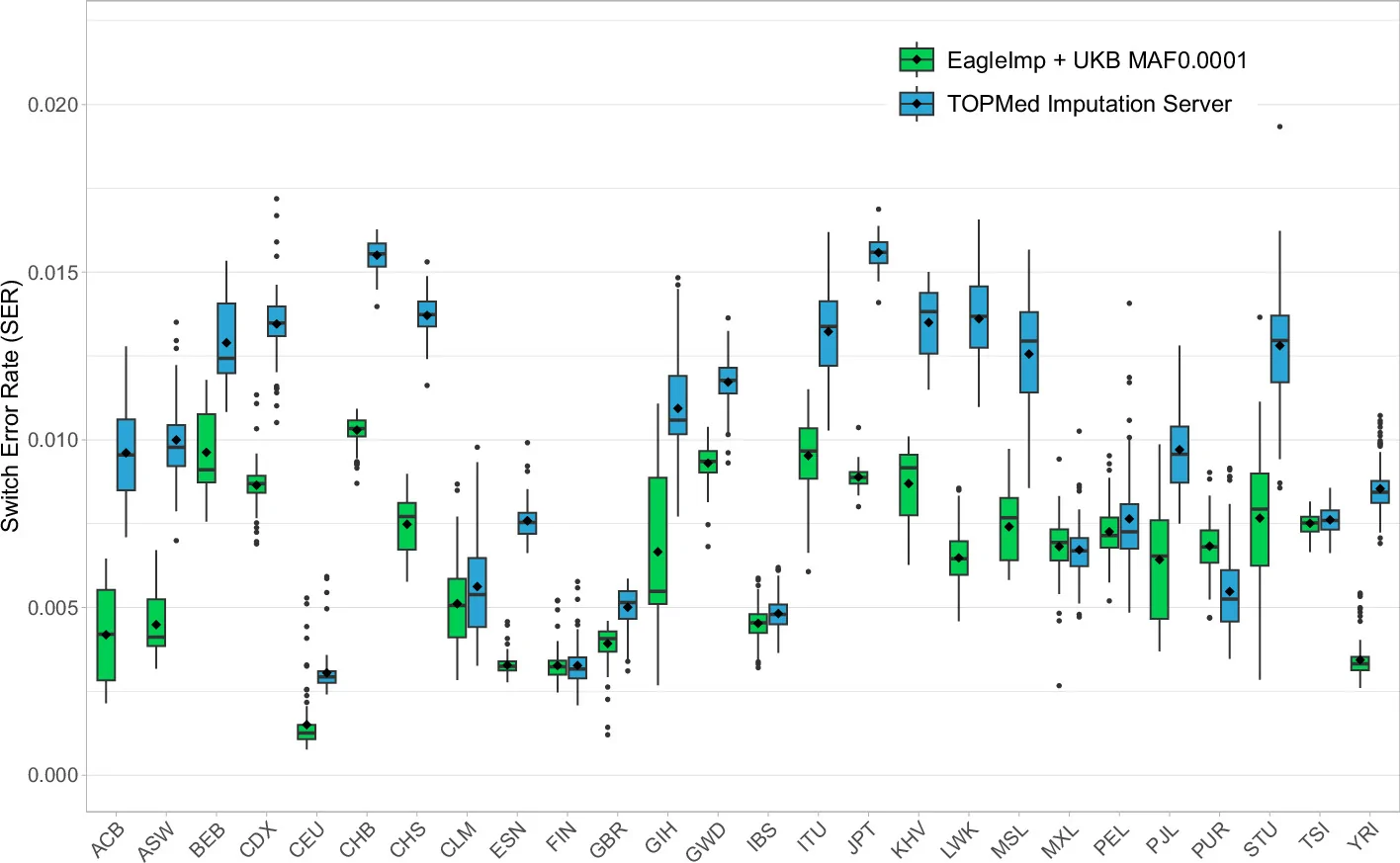
Box plots of the switch error rate (SER) for the 26 subpopulation benchmark datasets from the 1000 Genomes Project imputed with EagleImp and the rpMAF-filtered UK Biobank reference panel (UKB MAF0.0001), compared with TOPMed. SER is measured to the 1000 Genome reference (ground truth) and is based on all typed (input) variants shared with the TOPMed r3 reference and the 1000 Genomes reference. The number of shared variants is *n* = 540,537. The box shows the inner quartiles, and the whiskers indicate 1.5× the interquartile range. The bar indicates the median, and the mean is represented by the black diamond. Source data

For each imputation run of the 5 superpopulations and the 26 subpopulations, we calculated SER to assess preimputation phasing quality as well as MAE and correlation *r*^2^ for imputed genotypes across four rpMAF range categories. The complete UKB reference panel showed lower SERs than the TOPMed r3 panel for all superpopulations except AMR. Across the 26 subpopulations, 25 showed lower SERs with the UKB panel, with one subpopulation (PUR) showing no difference in the SER, while the TOPMed r3 panel yielded lower SERs in two cases (MXL and PUR). See Extended Data Figs. 5–8 and Supplementary Table 6 for details.

**Fig. 2:**
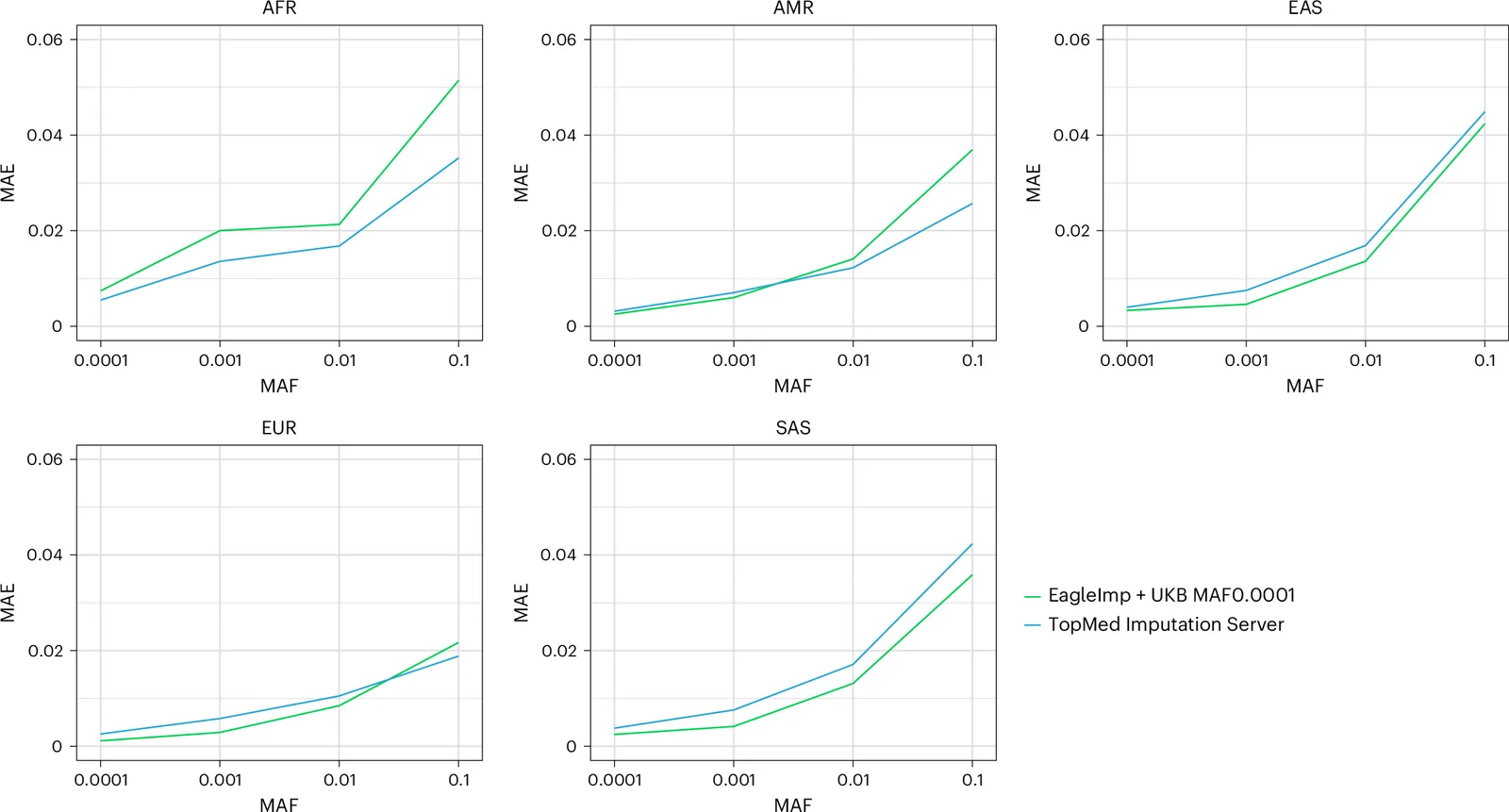
MAE imputation quality across 1000 Genomes superpopulations. MAE imputation quality results for the five superpopulations from the 1000 Genomes Project based on the 1000 Genomes reference panel ground truth, stratified by four rpMAF range categories (0.01%, 0.1%), (0.1%, 1%), (1%, 10%), (10%, 50%) and imputed by EagleImp with the rpMAF-filtered UKB reference panel (UKB MAF0.0001; green lines) and the TOPMed Imputation Server (TOPMed r3 panel; blue lines). MAE calculation is based on *n* = 30,098,506 variants shared among the UKB MAF0.0001, the TOPMed r3 panel and the 1000 Genomes Project panel. Source data

MAE-based analyses showed that the complete UKB reference panel outperformed the TOPMed r3 panel for all rpMAF range categories in the SAS and EAS superpopulations, for rpMAF range (0.01%, 0.1%) and (0.1%, 1%) in EUR and for rpMAF range (0.01%, 0.1%) in AMR (with similar performance for (0.1%, 1%)); the TOPMed r3 panel performed better across all rpMAF range categories in AFR. The rpMAF-filtered UKB reference panel showed the same overall result with slightly improved performance, including lower MAE than the TOPMed r3 panel for AMR in (0.1%, 1%). Subpopulation-level MAE results varied by reference panel, population and rpMAF range. Across the 26 subpopulations, the rpMAF-filtered UKB reference panel showed lower MAE across all rpMAF range categories for 12 populations, the TOPMed r3 panel for 6 populations and mixed results across 8 populations depending on rpMAF range category. MAE values, particularly in the lowest rpMAF range category, also reflect the predominance of major-allele genotypes in small target datasets, which should be considered when interpreting absolute differences across rpMAF categories, whereas our analyses primarily focus on relative performance differences between reference panels within the same rpMAF category. See Fig. 2, Supplementary Figs. 5–7 and Supplementary Tables 7 and 8 for details.

A commonly used alternative imputation metric is the squared Pearson correlation coefficient *r*^2^ between imputed allele dosages and masked ground-truth genotypes. However, due to its instability in small cohorts and its strong dependence on the estimation method (Supplementary Note 5, Supplementary Figs. 8–15 and Supplementary Tables 9–12), we do not interpret small numerical differences in correlation *r*^2^ as meaningful indicators of relative panel performance.

We repeated the benchmark using four real-world GWAS case-control datasets from Germany (3,351 samples), Italy (2,090 samples), Norway (324 samples) and Spain (1,725 samples) from refs. ^3,4^. As before, using the rpMAF-filtered UKB reference panel did not reduce the estimated imputation *R*^2^ (Supplementary Fig. 16), and the complete UKB panel improved imputation quality over the TOPMed r3 panel across the entire allele frequency spectrum (Supplementary Fig. 17). Figure 1 summarizes estimated *R*^2^ values and percentage improvements for the 5 superpopulations, the 26 subpopulations and the 4 real-world GWAS case-control datasets across three rpMAF thresholds.

**Fig. 3:**
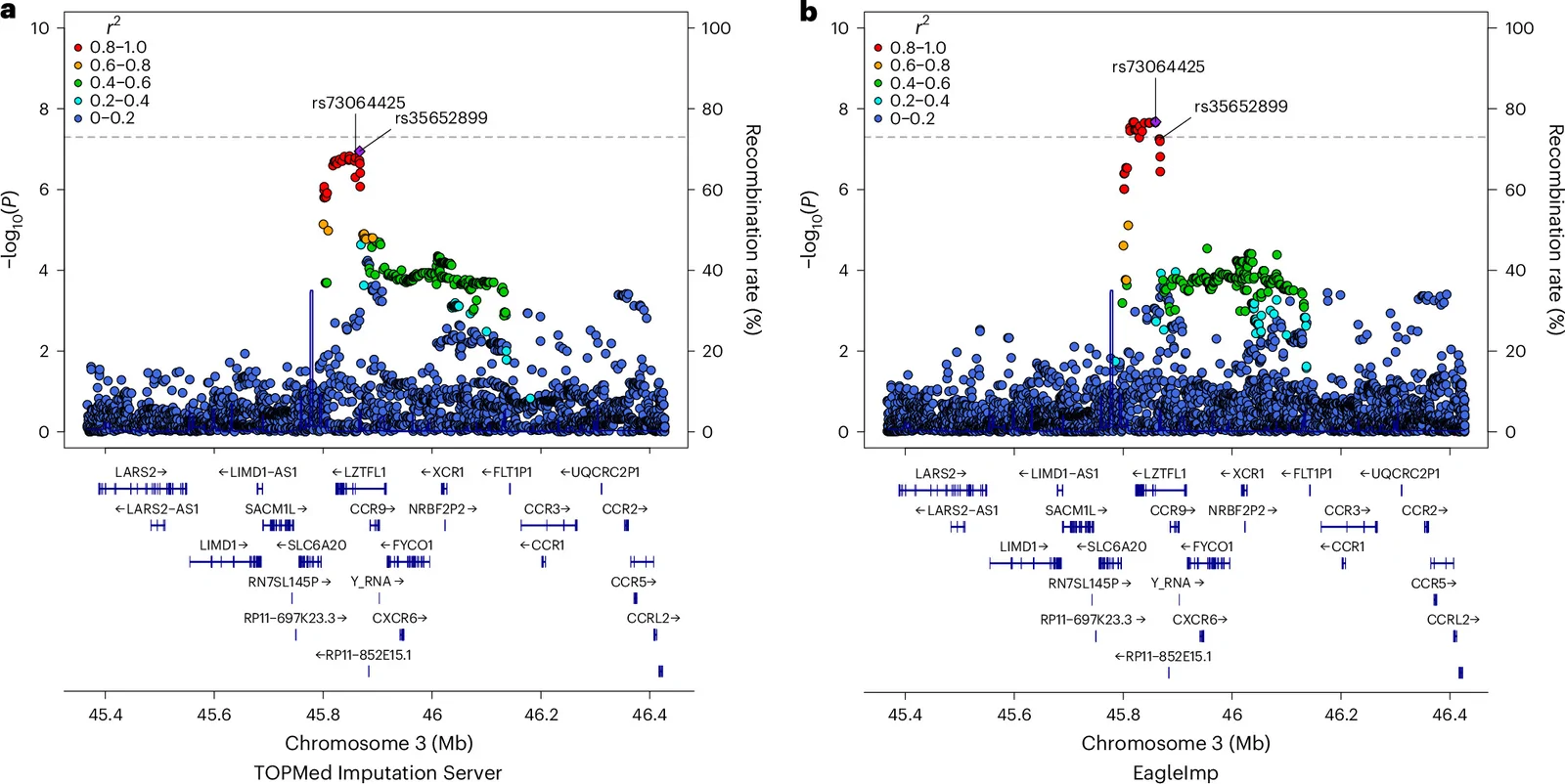
Improved COVID-19 risk locus association through EagleImp imputation. Regional association plots illustrating the improvement of association testing through improved imputation quality for common variants (MAF ≥ 1%) at the most strongly associated COVID-19 risk locus (3p21.31) using the COVID-19 GWAS dataset Italy (original data from ref. ^3^), indicating that imputation of common variants with the TOPMed Imputation Server is still not saturated in European populations. **a**, Imputation with the TOPMed Imputation Server (TOPMed reference panel). **b**, Imputation with EagleImp (rpMAF-filtered UKB reference panel). Imputed variants were filtered for reported estimated *R*^2^ ≥ 0.6 and MAF ≥ 1% for direct comparison, as performed in ref. ^3^. The dashed line represents the genome-wide association significance threshold at *P* = 5 × 10^−8^, with the Italian GWAS dataset showing a genome-wide significant association result when performing EagleImp’s UKB reference panel imputation. Highlighted variants are rs73064425 (lead SNP of the EagleImp imputation) and rs35652899 (lead SNP of the TOPMed Imputation Server imputation). The association signal improvement correlates with the observed improvement in estimated *R*^2^ of the Italy dataset shown in Fig. 1. The new lead SNP rs73064425 from the EagleImp imputation is also the lead SNP identified in the larger GenOMICC GWAS in 2,244 critically ill patients with COVID-19 from 208 UK intensive care units^9^, in the GenOMICC follow-up WGS study in 7,491 critically ill individuals with COVID-19 versus 48,400 controls^10^ and in the GWAS of 2,184 severely ill cases with COVID-19 versus 689,620 COVID-19-negative or unknown population controls^11^. LD analysis showed that the established lead variant rs73064425 is only in moderate LD with rs35652899 (LD *r*^2^ = 0.6625; lead SNP from TOPMed Imputation Server imputation), further indicating an improvement in imputation quality with EagleImp over the TOPMed Imputation Server. A Bayesian fine-mapping analysis using the SuSiE software^14^ (Methods) for the Italian GWAS results from the EagleImp imputation identified rs73064425 as the most likely causal variant in a credible set that contains the causal variant with a probability of ≥95% for locus 3p21.31 for critically ill patients with COVID-19 (Supplementary Table 13). On the other hand, the Bayesian fine-mapping analysis for the results of the TOPMed Imputation Server listed the established lead variant rs73064425 on the fourth rank of most likely causal variants, with a larger (less precise) credible set of associated variants (Supplementary Table 14). Single-marker association *P* values were obtained using two-sided additive logistic regression implemented in PLINK2, including ten principal components as covariates; no adjustment for multiple testing was applied. LD, linkage disequilibrium; GenOMICC, Genetics Of Mortality In Critical Care. Source data

Next, we examined the impact of UKB-based imputation on association testing and the detection of genome-wide significant association loci (*P* < 5 × 10^−8^). Because the real-world Italy GWAS dataset showed the greatest improvement in imputation quality, we repeated the GWAS study from ref. ^3^ (835 cases of severe COVID-19 with respiratory failure and 1,255 healthy controls; Methods) using imputed genotypes from EagleImp (rpMAF-filtered UKB reference panel) and from the TOPMed Imputation Server. Genome-wide association results were largely similar (see Manhattan and quantile–quantile plots in Supplementary Figs. 18 and 19), except at the well-established and most strongly associated COVID-19 susceptibility locus at 3p21.31 (also visible in the tail of the distribution of *P* values in the quantile–quantile plots). First, compared to TOPMed Imputation Server imputation (which was also performed in the original study in ref. ^3^), only the UKB-based imputation yielded a genome-wide significant association at 3p21.31, demonstrating improved common-variant imputation in this European cohort. Second, the new lead SNP rs73064425 replicated the signals reported in three independent GWAS on COVID-19 from refs. ^9–11^. Linkage disequilibrium and Bayesian fine-mapping analysis further confirmed improved resolution in imputation quality by EagleImp compared to the TOPMed Imputation Server (Fig. 3 and Supplementary Tables 13 and 14).

Because very large reference panels such as the UKB reference panels require additional compute resources, an increase in runtime and memory requirements for phasing and imputation is expected. We therefore measured and compared phasing and imputation runtimes (without queuing times) for the complete and rpMAF-filtered UKB reference panels (both using EagleImp and EagleImp-RAP) and the TOPMed r3 panel (TOPMed Imputation Server; Supplementary Note 4). The runtime comparison of EagleImp and EagleImp-RAP did not show considerable differences. On average across all 35 benchmark populations (Fig. 1), the runtime of the TOPMed Imputation Server with the TOPMed r3 panel was about twice as long as that of EagleImp and EagleImp-RAP with the complete UKB reference panel, although the UKB reference panel contains more than 3.6 times as many samples as the TOPMed r3 panel. For the rpMAF-filtered UKB reference panel, we measured an average 4–13 times shorter runtime of EagleImp and EagleImp-RAP compared to the TOPMed Imputation Server. See Table 2 for all measured runtimes.

**Table 2:** Imputation runtimes for phasing and imputation using the UKB reference panels

Dataset	EagleImp standalone		EagleImp-RAP		TOPMed
	UKB	UKB	UKB	UKB	TOPMed r3
	complete	MAF0.0001	complete	MAF0.0001	
	hh:mm:ss	hh:mm:ss	hh:mm:ss	hh:mm:ss	hh:mm:ss
Datasets from the 1000 Genomes Project^13^ superpopulations					
AFR	01:09:03	00:29:49	01:27:00	00:38:00	02:34:55
AMR	01:01:16	00:26:26	01:14:00	00:34:00	02:03:02
EAS	01:02:31	00:26:48	01:20:00	00:37:00	02:25:52
EUR	01:17:33	00:29:53	01:25:00	00:35:00	02:01:16
SAS	01:06:31	00:27:37	01:18:00	00:34:00	02:09:50
Datasets from the 1000 Genomes Project^13^ subpopulations					
ACB	00:42:16	00:21:50	00:53:00	00:29:00	02:26:56
ASW	00:41:19	00:21:19	00:52:00	00:28:00	02:22:39
BEB	00:44:01	00:20:41	00:54:00	00:29:00	01:35:40
CDX	00:41:20	00:23:00	00:54:00	00:28:00	01:21:38
CEU	00:42:38	00:22:41	00:54:00	00:27:00	01:14:54
CHB	00:43:30	00:22:45	00:55:00	00:28:00	01:20:00
CHS	00:41:51	00:23:14	00:55:00	00:27:00	05:17:13
CLM	00:44:42	00:22:07	00:56:00	00:28:00	01:23:36
ESN	00:43:24	00:21:23	00:54:00	00:28:00	01:08:49
FIN	00:46:08	00:22:39	00:55:00	00:28:00	01:26:36
GBR	00:45:20	00:22:52	00:54:00	00:28:00	01:26:53
GIH	00:42:20	00:24:14	00:55:00	00:27:00	01:28:13
GWD	00:41:46	00:21:44	00:55:00	00:28:00	01:28:10
IBS	00:46:59	00:24:24	00:57:00	00:29:00	01:26:44
ITU	00:43:19	00:22:13	00:54:00	00:28:00	01:14:54
JPT	00:45:44	00:21:23	00:55:00	00:29:00	01:17:17
KHV	00:43:11	00:21:13	00:55:00	00:28:00	01:12:48
LWK	00:41:04	00:21:55	00:54:00	00:28:00	01:16:24
MSL	00:42:56	00:21:53	00:52:00	00:27:00	01:13:54
MXL	00:42:02	00:21:58	00:53:00	00:27:00	01:12:15
PEL	00:42:09	00:22:19	00:53:00	00:27:00	01:07:59
PJL	00:44:51	00:22:19	00:55:00	00:27:00	01:27:30
PUR	00:46:18	00:21:55	00:57:00	00:28:00	01:04:24
STU	00:42:05	00:22:48	00:53:00	00:27:00	01:18:30
TSI	00:47:27	00:24:39	00:59:00	00:28:00	01:12:43
YRI	00:43:13	00:21:40	00:53:00	00:27:00	01:10:13
Datasets from COVID-19 studies^3,4^					
Germany	06:56:03	01:35:13	05:23:00	01:21:00	13:12:32
Italy	04:52:13	01:08:34	03:36:00	01:08:00	08:19:25
Norway	01:14:25	00:25:04	01:17:00	00:31:00	05:34:34
Spain	04:08:37	00:58:01	03:07:00	00:57:00	06:26:50

Runtimes were measured for the complete (UKB complete, hg38) and MAF-filtered (UKB MAF0.0001, hg38) panels using EagleImp on the project’s secure standalone compute server and using EagleImp-RAP within the UKB-RAP. For comparison, runtimes of the TOPMed Imputation Server (TOPMed r3 panel) are included. Details of the runtime measurement procedures for all systems are provided in Supplementary Note 4. Descriptions and details of the benchmarks datasets can be found in Supplementary Table 3. Runtimes exclude queuing times.

Interestingly, a haplotype reference panel of 78,195 individuals from the Genomics England (GEL) study was recently published in ref. ^12^ used for imputation of GWAS data from the UKB. We were unable to include the GEL imputation reference panel in our benchmarks, as the GEL imputation reference panel is not publicly available and there is no publicly accessible GEL imputation server for direct use of the panel. An improvement observed in ref. ^12^ in phasing and imputation quality over the TOPMed Imputation Server with the TOPMed r2 panel for the Great Britain (GBR) subpopulation and the South Asian (SAS) population from the 1000 Genomes Project, but a decrease in phasing and imputation quality for the AFR, AMR, EAS and non-Finnish European populations for variants with MAF of ≥0.01%. Because our UKB reference panel imputation benchmarks with 35 study populations showed an overall improvement in imputation quality up to 24.39%, we believe that the UKB reference panel is the most suitable reference panel for imputing worldwide populations if no population-specific reference panel is available, as in the example of the GEL reference panel for the majority of the British population. We would like to point out that the TOPMed r3 panel, with a total of 244,838,777 reference panel-specific variants, has more reference panel-specific variants (with unknown allele frequencies) than the complete UKB reference panel with 161,298,331 panel-specific variants (with a minor allele count (MAC) of at least 4), which makes it possible to impute more study population-specific variants from non-European populations with the TOPMed r3 panel.

Through our reimputation benchmarks of published COVID-19 GWAS case-control datasets and subsequent association and fine-mapping analyses, we were able to show that imputation of common variants (MAF ≥ 1%) is not saturated in respect to imputation quality for European study populations, in contrast to the results of the imputation benchmarks using the GEL reference panel^12^. For European populations that are not well-represented in current imputation reference panels, we therefore recommend imputation with the UKB reference panel.

A limitation of our study is that for many populations worldwide there is still room for improvement in terms of imputation quality, especially for rare variants. The imputation quality benchmark measures MAE and correlation *r*^2^ based on a known ground truth, but it has advantages and disadvantages and may be inaccurate for low MAFs. And the average estimated imputation *R*^2^ for variants with a MAF of <0.01% is still low for most worldwide subpopulations. A global superpanel with millions of haplotypes or the availability of several population-specific reference panels worldwide through imputation servers or cloud-based platforms could help here in the future.

## Methods

This research has been conducted using the UKB Resource under application 139525. Ethical approval for COVID-19 GWAS data from the COVID-19 GWAS Group—all participants in the study provided written informed consent, and the study was approved by the ethics boards of the participating institutions in agreement with the Declaration of Helsinki principles.

This section summarizes the creation of the UKB haplotype reference panels and the association test of reimputed COVID-19 GWAS data, including subsequent Bayesian fine-mapping analysis. For details regarding the environmental setup and the conduction of imputation quality and runtime analyses, please refer to Supplementary Note 4.

### Phasing of whole-genome sequencing data from UKB

This section summarizes the process of creating the complete UKB reference panel. The populations represented in the UKB reference panels, as reported in ref. ^1^, are summarized in Supplementary Table 2. Command calls and technical details can be found in Supplementary Note 3.

#### Data preparation of whole-genome sequencing data from UKB

For the UKB reference panel, we used the GraphTyper population-level whole-genome sequencing (WGS) variants (500,000 release) in pVCF format. The data are available in the UKB-RAP (https://ukbiobank.dnanexus.com) as packed .vcf.gz files in unphased chunks of 20 kbp. The total number of files is 151,561, with an average file size of 9.96 GB, for a total of 1509.29 TB. We created a custom tool, vcffilter, written in C*,* to extract variant positions, alleles, the *FILTER* and *INFO* columns and the genotypes. We used the UKB-RAP to process all pVCF files with this tool, resulting in the same number of files but with an average file size of only 27.86 MB and a total size of 4123.70 GB. We then downloaded the generated files from the UKB-RAP to our own computing resources for further processing. To restore a valid VCF from our custom download format, we used a simple pendant of our C++ extraction tool. Both tools are available in the Vcffilter toolset at https://github.com/ikmb/vcffilter. Furthermore, the restoration tool performs custom filtering and converts multi-allelic variants to several biallelics on the fly, which is faster than first restoring a complete VCF file and then applying BCFtools^15^ for filtering and conversion. In particular, we converted all multi-allelic variants to biallelics as if BCFtools was applied with BCFtools norm -m, and, as suggested in ref. ^16^ for quality control, we filtered out all variants with (1) a value other than PASS in the FILTER column, (2) a missingness rate of >10% and (3) an alternative allele score of <0.8. Furthermore, we created two batches of files by (1) keeping only variants with a MAF of ≥0.001 and (2) keeping variants with a MAC of ≥4.

#### Phasing of WGS data from UKB

The phasing process using SHAPEIT5^17^ is divided into two parts. First, a scaffold is created by phasing only common variants with MAF ≥ 0.001, that is, our first batch, with the tool phase_common. Second, the tool phase_rare is used to phase the rare variants from our second batch, but using the phased scaffold from the first step.

For phasing the common variants, chunks were created of size around 20–30 Mbp for each chromosome, including an overlap of around 2 Mbp, by simply concatenating the corresponding 20-kbp-chunk files using BCFtools with the --naive-force option. We used the chunk limits in correspondence with the chunks used by Delaneau in https://github.com/odelaneau/shapeit/tree/main/tasks/phasingUKB_200k_release/autosomes/chunks.tgz. Phasing is then conducted by calling phase_common with the default options. As the chunks were phased separately and the phases could switch between chunks, a subsequent ligation step is necessary to create the final scaffold. We used the ligation option -l provided by BCFtools to ligate and merge in one step.

For phasing the rare variants, we used the second batch of our input files with variants with MAC ≥ 4. Now, we created batches with a size of approximately 5 Mbp and without overlaps. We used phase_rare from SHAPEIT5 with the declared input region and scaffold to conduct the phasing.

#### Creating Qref reference panel files

We used BCFtools with the --naive option to concatenate the phased chunks from the previous step. Ligation, as for generating the scaffold, is not required here as the phases are already in concordance with the previously generated scaffold used for phasing the rare variants. Next, we converted the generated phased chromosome files to our Qref format used in EagleImp^2^ for much faster reference panel loading during imputation (compared to loading a reference panel from .bcf or .vcf.gz format).

#### Special treatment of chromosome X

Male samples are encoded as diploid on chromosome X in the UKB GraphTyper data. However, diploid calls are allowed in the PAR1 and PAR2 regions (PAR1 and PAR2 of the X and Y chromosome pair recombine during meiosis), but have to be haploid in the non-PAR region (chrX: 2,781,480–155,701,382). To take account of this, we handled these three regions separately. First, in particular, we phased the PAR1/PAR2 regions and also the non-PAR region separately according to the procedure described above. This was possible because (haploid) males were encoded as homozygous diploid in the non-PAR region. Second, we fixed the ploidy afterward for the final phased output using the standard BCFtools plugin fixploidy for each chunk. As this step potentially affects ACs, we applied the BCFtools plugin fill-tags to correct the AC (INFO/AC) and allele number (INFO/AN) fields, and finally filtered again for MAC ≥ 4 using BCFtools filter to stay consistent with our original demand on the panel. Finally, three separate reference panel files in Qref format for chromosome X (one for each PAR1/non-PAR/PAR2 region) were generated as described above.

### rpMAF-filtered reference panel UKB MAF0.0001

Generally, imputation is often conducted as a preliminary step in a GWAS analysis. To reduce noise in the association output, usually prefiltering for high-quality imputed variants is done. This is achieved by filtering using an estimated *R*^2^ threshold of, for example, 0.6 or higher. Our benchmarks showed that regardless of the input dataset, the mean estimated *R*^2^ of variants with an rpMAF of <0.0001 is far below 0.6; thus, it is unlikely to generate *R*^2^ values higher than 0.6 for such variants and their contribution to the GWAS analysis is rather small. Consequently, the use of the complete UKB reference panel in GWAS is only required if an analysis of rare variants is desired and a sufficient GWAS sample size is available (for sufficient statistical power). For a typical GWAS analysis of common variants, we therefore decided to provide a second reference panel with variants with MAF ≥ 0.0001 for a much faster imputation. We thus used our previously generated phased chromosome files, filtered for MAF ≥ 0.0001 and converted the output to the Qref format again. This second panel can be selected in EagleImp to conduct faster imputation for common GWAS analyses without considerable loss of imputation quality. We have proven the persistent quality with the runtime advantages in our benchmarks. An exemplary GWAS conducted with the rpMAF-filtered UKB reference panel is presented in Fig. 3 and described below.

### Association testing of reimputed GWAS data

To demonstrate the capabilities of the UKB panel, we repeated a GWAS of severe COVID-19 with respiratory failure with original data from ref. ^3^ for 835 Italian cases with severe respiratory failure and 1,255 Italian controls. We reimputed the Italian dataset with EagleImp using the rpMAF-filtered UKB panel (UKB MAF0.0001) with the phasing default parameter *K* = 10, 000. We used the BIGwas^18^ association software pipeline with PLINK2 (ref. ^19^) and ten principal components from principal component analysis to generate single-marker summary statistics from imputed data. The summary statistics were further filtered with an estimation *R*^2^ value of 0.6 and a MAF of 0.01 (1%) for comparability with the results of the original study.

### Bayesian fine-mapping analysis of reimputed GWAS data

Statistical fine-mapping analysis was performed with the software SuSiE^14^ at locus 3p21.31 for GWAS association results from the reimputed Italian GWAS dataset. SuSiE, using the susie_rss method, calculated posterior inclusion probabilities for each SNP at locus 3p21.31 to determine the 95% credible set of SNPs, assuming one credible set of variants, thus generating the minimum set of variants containing the causal variant with ≥95% certainty.

### Implementation of EagleImp-RAP

EagleImp-RAP was implemented as a DNAnexus applet on the UKB-RAP, following the DNAnexus developer guidelines at https://documentation.dnanexus.com/developer/apps/intro-to-building-apps. Using the dx-app-wizard from the DXtools package, we generated a standardized UKB-RAP applet structure containing the execution bash script, a .json configuration file and a template directory structure for required binaries and libraries. EagleImp, htslib and BCFtools were compiled for the UKB-RAP target environment. We also added a precompiled version of Intel’s libtbb (as required by EagleImp). The execution script handles argument parsing, retrieval and indexing of input files, and downloading the selected UKB reference panel from a predefined location within UKB-RAP. After running EagleImp on the acquired virtual machine, the script collects and returns all output files to the user’s project space. Chromosome X input files require special handling, as male samples are haploid in non-PAR regions and diploid in PAR1/PAR2. The applet therefore splits chromosome X into PAR1, non-PAR and PAR2 segments, processes each region separately using the appropriate reference panel, and merges the results into a unified output file.

### Reporting summary

Further information on research design is available in the Nature Portfolio Reporting Summary linked to this article.

## Online content

Any methods, additional references, Nature Portfolio reporting summaries, source data, extended data, supplementary information, acknowledgements, peer review information; details of author contributions and competing interests; and statements of data and code availability are available at https://doi.org/10.1038/s41588-026-02819-0.

## Supplementary information

Supplementary Information Supplementary Notes 1–5, Figs. 1–19 and Tables 1–14.

Reporting Summary

Peer Review File

## Source data

Source Data Fig. 1 Estimated *R*^2^ for all benchmarked populations.

Source Data Fig. 2 Statistical source data (MAE).

Source Data Fig. 3 PLINK summary statistics from association analysis.

Source Data Extended Data Fig. 3 Estimated *R*^2^ for all 1000 Genomes superpopulations (complete UKB panel versus MAF-filtered UKB panel).

Source Data Extended Data Fig. 4 Estimated *R*^2^ for all 1000 Genomes superpopulations (complete UKB panel versus TOPMed).

Source Data Extended Data Fig. 5 Statistical source data for SER (1000 Genomes superpopulations, complete UKB panel versus TOPMed).

Source Data Extended Data Fig. 6 Statistical source data for SER (1000 Genomes superpopulations, MAF-filtered UKB panel versus TOPMed).

Source Data Extended Data Fig. 7 Statistical source data for SER (1000 Genomes subpopulations, complete UKB panel versus TOPMed).

Source Data Extended Data Fig. 8 Statistical source data for SER (1000 Genomes subpopulations, MAF-filtered UKB panel versus TOPMed).

## Data Availability

UKB-based imputation analyses were performed exclusively within secure compute environments approved for UKB project 139525. The complete UKB reference panel (UKB complete) and the MAF-filtered version (UKB MAF0.0001) were processed and benchmarked on the project’s institutional secure compute server, which corresponds to the standalone back-end server of EagleImp-Web but is not accessible to external users. To demonstrate reproducibility within UKB’s cloud infrastructure, we additionally implemented EagleImp-RAP, a version of EagleImp within the UKB-RAP, enabling benchmark runs directly within UKB-RAP. The benchmark datasets created from 1000 Genomes data are available for download at https://hybridcomputing.ikmb.uni-kiel.de/imputation-benchmark. These include all superpopulation and subpopulation datasets from the 1000 Genomes Project^13^, which have been reduced to Illumina Global Screening Array-chip variants used for our benchmarks. The COVID-19 GWAS benchmark datasets are available with conditions and only on request to the COVID-19 GWAS Group. Benchmarking results are presented in Figs. 1–3, Extended Data Figs. 3–8, Supplementary Figs. 5–19, Table 2 and Supplementary Tables 4–14. Source data are provided with this paper.

## Code availability

The EagleImp software used for standalone phasing and imputation is freely available under the GPL (v3) license at https://github.com/ikmb/eagleimp and was published in ref. ^2^. The source code for creating the EagleImp-RAP applet (under the GPL (v3) license), including a usage example, is available at https://github.com/ikmb/eagleimp-rap (https://doi.org/10.5281/zenodo.22688278). Our custom C/C^++^ tools for fast data extraction from VCF files and VCF restoration are available for download in the Vcffilter project under the GPL (v3) license at https://github.com/ikmb/vcffilter.
