# Supplementary material for: A haplotype reference panel constructed from 490,319 UK Biobank genomes improves genotype imputation for global populations
Source: Nat Genet. 2026 Oct 30;58(11):2778–83. doi: 10.1038/s41588-026-02819-0 (PMC13754612; doi:10.1038/s41588-026-02819-0)
Supplement: Supplementary file 2 [file 41588_2026_2819_MOESM2_ESM.pdf]

nature portfolio

Corresponding author(s): Lars Wienbrandt, David Ellinghaus

Last updated by author(s): 15.09.2026

Reporting Summary

Nature Portfolio wishes to improve the reproducibility of the work that we publish. This form provides structure for consistency and transparency in reporting. For further information on Nature Portfolio policies, see our Editorial Policies and the Editorial Policy Checklist.

Statistics

For all statistical analyses, confirm that the following items are present in the figure legend, table legend, main text, or Methods section.

n/a	Confirmed	
☐	☒	The exact sample size (n) for each experimental group/condition, given as a discrete number and unit of measurement
☐	☒	A statement on whether measurements were taken from distinct samples or whether the same sample was measured repeatedly
☐	☒	The statistical test(s) used AND whether they are one- or two-sided<br>*Only common tests should be described solely by name; describe more complex techniques in the Methods section.*
☐	☒	A description of all covariates tested
☐	☒	A description of any assumptions or corrections, such as tests of normality and adjustment for multiple comparisons
☐	☒	A full description of the statistical parameters including central tendency (e.g. means) or other basic estimates (e.g. regression coefficient) AND variation (e.g. standard deviation) or associated estimates of uncertainty (e.g. confidence intervals)
☐	☒	For null hypothesis testing, the test statistic (e.g. F, t, r) with confidence intervals, effect sizes, degrees of freedom and P value noted<br>*Give P values as exact values whenever suitable.*
☐	☒	For Bayesian analysis, information on the choice of priors and Markov chain Monte Carlo settings
☒	☐	For hierarchical and complex designs, identification of the appropriate level for tests and full reporting of outcomes
☐	☒	Estimates of effect sizes (e.g. Cohen's d, Pearson's r), indicating how they were calculated

Our web collection on statistics for biologists contains articles on many of the points above.

Software and code

Policy information about availability of computer code

Data collection	UKB-based imputation analyses were performed exclusively within secure compute environments approved for UK Biobank Project 139525. The complete UKB reference panel (UKB complete) and the MAF-filtered version (UKB MAF0.0001) were processed and benchmarked on the project's institutional secure compute server, which corresponds to the standalone backend server of EagleImp-Web but is not accessible to external users. To demonstrate reproducibility within UK Biobank's cloud infrastructure, we additionally implemented EagleImp-RAP, a version of EagleImp within the UK Biobank Research Analysis Platform (UKB-RAP), enabling benchmark runs directly within UKB-RAP. The benchmark datasets created from 1000 Genomes data are available for download at https://hybridcomputing.ikmb.uni-kiel.de/imputation-benchmark. These include all super population and subpopulation datasets from the 1000 Genomes Project, which have been reduced to Illumina GSA-chip variants used for our benchmarks. GWAS data from the COVID-19 GWAS Group: All study participants provided written informed consent, and the study was approved by the ethics boards of the participating institutions in agreement with the Declaration of Helsinki principles. The authors would like to thank the COVID-19 GWAS Group for the use of the COVID-19 GWAS data for the benchmark.
Data analysis	The EagleImp software used for standalone phasing and imputation is freely available under the GPL v3 license at https://github.com/ikmb/eagleimp. The source code for creating the EagleImp-RAP applet (under the GPL v3 license) including a usage example is available at https://github.com/ikmb/eagleimp-rap (Zenodo DOI: 10.5281/zenodo.22688278). Our custom C/C++ tools for fast data extraction from VCF files and VCF restoration are available for download in the Vcffilter project under the GPL v3 license at https://github.com/ikmb/vcffilter.

For manuscripts utilizing custom algorithms or software that are central to the research but not yet described in published literature, software must be made available to editors and reviewers. We strongly encourage code deposition in a community repository (e.g. GitHub). See the Nature Portfolio guidelines for submitting code & software for further information.

Data

Policy information about availability of data

All manuscripts must include a data availability statement. This statement should provide the following information, where applicable:

- Accession codes, unique identifiers, or web links for publicly available datasets
- A description of any restrictions on data availability
- For clinical datasets or third party data, please ensure that the statement adheres to our policy

To build the UK Biobank imputation reference panels, we extracted and packed genotype information from all 151,561 available Graphtyper pVCF format files on the UK Biobank Research Analysis Platform (RAP), originally comprising about 1509.25 TB of data. The data is available in the UK Biobank Research Access Platform (RAP) (https://ukbiobank.dnanexus.com) as packed .vcf.gz files in unphased chunks of 20 kbp. The total number of files is 151,561 319 with an average file size of 9.96GB which sums up to a total of 1509.29TB. We downloaded the extracted genotype information (reduced to 4123.70 GB) from the RAP (Online Methods) and further processed it using our compute resources at the University Medical Center Schleswig-Holstein (UKSH) in Kiel, Germany, in accordance with the data processing and downloading regulations and security measures set out in UK Biobank's Material Transfer Agreement (MTA). This research has been conducted using the UK Biobank Resource under application number 139525. The benchmark datasets created from 1000 Genomes data are available for download at https://hybridcomputing.ikmb.uni-kiel.de/imputation-benchmark. These include all superpopulation and subpopulation datasets from the 1000 Genomes Project, which have been reduced to Illumina GSA-chip variants used for our benchmarks.

Research involving human participants, their data, or biological material

Policy information about studies with human participants or human data. See also policy information about sex, gender (identity/presentation), and sexual orientation and race, ethnicity and racism.

Reporting on sex and gender	Populations in the UKB panel as reported in Extended Data Table 3 of (Bycroft et al. Nature, 562(7726), 203–209) (participants self-reported their ethnic background in a questionnaire). 2,131 additional individuals present in the Graphtyper data and missing in the table were added to the Other/Unknown group. See also Supplementary Table 2. biological sex: 265,816 females and 224,503 males.
Reporting on race, ethnicity, or other socially relevant groupings	Populations in the UKB panel as reported in Extended Data Table 3 of (Bycroft et al. Nature, 562(7726), 203–209) (participants self-reported their ethnic background in a questionnaire). 2,131 additional individuals present in the Graphtyper data and missing in the table were added to the Other/Unknown group. See also Supplementary Table 2.
Population characteristics	Populations in the UKB panel as reported in Extended Data Table 3 of (Bycroft et al. Nature, 562(7726), 203–209) (participants self-reported their ethnic background in a questionnaire). 2,131 additional individuals present in the Graphtyper data and missing in the table were added to the Other/Unknown group. See also Supplementary Table 2.
Recruitment	Populations in the UKB panel as reported in Extended Data Table 3 of (Bycroft et al. Nature, 562(7726), 203–209) (participants self-reported their ethnic background in a questionnaire). 2,131 additional individuals present in the Graphtyper data and missing in the table were added to the Other/Unknown group. See also Supplementary Table 2.
Ethics oversight	This research has been conducted using the UK Biobank Resource under application number 139525.

Note that full information on the approval of the study protocol must also be provided in the manuscript.

Field-specific reporting

Please select the one below that is the best fit for your research. If you are not sure, read the appropriate sections before making your selection.

☒ Life sciences ☐ Behavioural & social sciences ☐ Ecological, evolutionary & environmental sciences

For a reference copy of the document with all sections, see nature.com/documents/nr-reporting-summary-flat.pdf

Life sciences study design

All studies must disclose on these points even when the disclosure is negative.

Sample size	In this study, we constructed a whole-genome haplotype reference panel from 490,319 individuals and more than 361 million genetic variants from UK Biobank. For the UKB reference panel we used the GraphTyper population level whole-genome sequencing (WGS) variants (500k release) in pVCF format. The data is available in the UK Biobank Research Access Platform (RAP) (https://ukbiobank.dnanexus.com) as packed .vcf.gz files in unphased chunks of 20 kbp. The total number of files is 151,561 319 with an average file size of 9.96GB which sums up to a total of 1509.29TB.
Data exclusions	not applicable
Replication	The benchmark datasets created from 1000 Genomes data are available for download at https://hybridcomputing.ikmb.uni-kiel.de/imputation-benchmark. These include all superpopulation and subpopulation datasets from the 1000 Genomes Project (https://

ftp.1000genomes.ebi.ac.uk/vol1/ftp/release/20130502/), which have been reduced to Illumina GSA-chip variants used for our benchmarks. GWAS data from the COVID-19 GWAS Group: All study participants provided written informed consent, and the study was approved by the ethics boards of the participating institutions in agreement with the Declaration of Helsinki principles. The authors would like to thank the COVID-19 GWAS Group for the use of the COVID-19 GWAS data for the benchmark.

Randomization	GWAS data from the COVID-19 GWAS Group: we used original case-control samples as published in (Ellinghaus, D. et al. The New England Journal of Medicine, 383(16), 1522–1534. and Degenhardt, F. et al. Hum. Mol. Genet., 31(23), 3945–3966.). Germany: 241 COVID19 cases with severe respiratory failure + 3,110 controls. Italy: 835 COVID19 cases with severe respiratory failure + 1,255 controls. Norway: 62 COVID19 cases with severe respiratory failure + 262 controls. Spain: 775 COVID19 cases with severe respiratory failure + 950 controls.
Blinding	not applicable

Reporting for specific materials, systems and methods

We require information from authors about some types of materials, experimental systems and methods used in many studies. Here, indicate whether each material, system or method listed is relevant to your study. If you are not sure if a list item applies to your research, read the appropriate section before selecting a response.

Materials & experimental systems

n/a	Involved in the study
☒	☐ Antibodies
☒	☐ Eukaryotic cell lines
☒	☐ Palaeontology and archaeology
☒	☐ Animals and other organisms
☒	☐ Clinical data
☒	☐ Dual use research of concern
☒	☐ Plants

Methods

n/a	Involved in the study
☒	☐ ChIP-seq
☒	☐ Flow cytometry
☒	☐ MRI-based neuroimaging

Plants

Seed stocks	*Report on the source of all seed stocks or other plant material used. If applicable, state the seed stock centre and catalogue number. If plant specimens were collected from the field, describe the collection location, date and sampling procedures.*
Novel plant genotypes	*Describe the methods by which all novel plant genotypes were produced. This includes those generated by transgenic approaches, gene editing, chemical/radiation-based mutagenesis and hybridization. For transgenic lines, describe the transformation method, the number of independent lines analyzed and the generation upon which experiments were performed. For gene-edited lines, describe the editor used, the endogenous sequence targeted for editing, the targeting guide RNA sequence (if applicable) and how the editor was applied.*
Authentication	*Describe any authentication procedures for each seed stock used or novel genotype generated. Describe any experiments used to assess the effect of a mutation and, where applicable, how potential secondary effects (e.g. second site T-DNA insertions, mosiacism, off-target gene editing) were examined.*
