# Supplementary material for: A haplotype reference panel constructed from 490,319 UK Biobank genomes improves genotype imputation for global populations
Source: Nat Genet. 2026 Oct 30;58(11):2778–83. doi: 10.1038/s41588-026-02819-0 (PMC13754612; doi:10.1038/s41588-026-02819-0)
Supplement: Supplementary file 3 [file 41588_2026_2819_MOESM3_ESM.pdf]

nature portfolio

Peer Review File

A haplotype reference panel constructed from 490,319 UK Biobank genomes improves genotype imputation for global populations

Corresponding Author: Professor David Ellinghaus

Version 0:

Decision Letter:

28th May 2025

Dear David,

Your Letter entitled "A haplotype reference panel constructed from 490,508 UK Biobank genomes improves genotype imputation for global populations" has been seen by three reviewers, whose comments are included below. While they find your work of potential interest, they have raised substantial concerns that, in our view, are sufficiently important that they preclude publication of the work in Nature Genetics, at least in its present form.

In particular, Reviewer #1 has highlighted key concerns related to the legal and regulatory basis for conducting this work, and Reviewers #2 and #3 have raised overlapping concerns about the metrics used to assess imputation quality.

Should further documentation and additional analyses allow you to fully address these criticisms, we would be willing to consider an appeal of our decision (unless, of course, something similar has by then been accepted at Nature Genetics or appeared elsewhere). This includes submission or publication of a portion of this work someplace else.

We hope you understand that, until we have considered your responses to these concerns and read the revised manuscript in its entirety, we cannot promise that it will be sent back out for peer review.

If you are interested in attempting to revise this manuscript for resubmission to Nature Genetics in the future, please contact me to discuss a potential appeal. Otherwise, we hope that you find these comments helpful when preparing your manuscript for resubmission elsewhere.

Sincerely,
Kyle

Kyle Vogan, PhD
Senior Editor
Nature Genetics
https://orcid.org/0000-0001-9565-9665

Referee expertise:

Referee #1: Genetics, statistical methods, imputation

Referee #2: Genetics, statistical methods, imputation

Referee #3: Genetics, statistical methods, imputation

Reviewers' Comments:

Reviewer #1 (Remarks to the Author):

The authors present a haplotype reference panel constructed from 490,508 UK Biobank genomes that demonstrates improved imputation quality across diverse populations. However, my major concern relates to the legal and regulatory basis for constructing this reference panel. According to the authors, this research "has been conducted using the UK Biobank Resource under application number 139525." Based on publicly available information about this application (https://www.ukbiobank.ac.uk/enable-your-research/approved-research/integrative-multi-omics-and-computational-phenotyping-for-precision-medicine-in-inflammatory-bowel-disease), it is unclear whether the approved scope includes the construction of a reference panel hosted outside UK Biobank's secure infrastructure.

Furthermore, the authors employed an unconventional approach to data acquisition by downloading 151,561 unphased chunks of 20 kbp. This methodology appears to circumvent UK Biobank's standard data access protocols, as UK Biobank has implemented download limitations specifically to prevent bulk data extraction of this nature. Such an approach raises questions about compliance with approved data access terms and adherence to UK Biobank's data governance framework.

Given these concerns, I believe it is essential for the authors to provide comprehensive documentation demonstrating that their approved application specifically covers reference panel construction and external hosting, along with evidence of any special permissions or exemptions obtained for their data extraction methodology (for example, see UK Biobank's data access exemption procedures: https://community.ukbiobank.ac.uk/hc/en-gb/articles/23418486279325-Applying-for-a-data-access-exemption). Without such documentation, the regulatory foundation for this otherwise scientifically valuable work remains questionable.

Reviewer #2 (Remarks to the Author):

In this manuscript, Wienbrandt et al presents a valuable resource, an imputation reference panel constructed from ~half million UK Biobank individuals. The manuscript is brief yet informative. The resource has high potential to benefit the large scientific community. There are many additional merits of the manuscript, including evaluation of the impact on downstream analyses such as association testing and fine-mapping, as well as the inclusion of chromosome X.

The biggest concern with the current analyses is that the evaluations are based on ESTIMATED imputation quality rather than TRUE imputation quality. Reference panels leading to better ESTIMATED imputation quality may simply reflect an over-estimation or mis-calibration rather than truly better quality. For example, a reference panel with low haplotype diversity due to smaller sample sizes or less heterogenous populations can result in unwarranted high confidence in imputation output. In addition, the estimated imputation quality metric has also been shown to deviate more from true quality for lower frequency variants, which larger reference panels such as the one presented here are most valuable. Therefore, the evidence provided in the current manuscript fails to support the value of the reference panel. More precisely, it is impossible to judge the value of the constructed reference panel with the results based on estimated imputation quality. The concern, however, can be rather easily addressed by masking some variants and evaluating the true quality. A few more specific comments are listed below.

L145-147: instead of using cumulative categories, disjoint MAF categories (i.e., 0.01-0.1%, 0.1-1%, >= 1%) are recommended to avoid inflated imputation quality estimate by including common variants (which are well imputed even with earlier reference panels such as 1KG).

In the manuscript, the authors only compared variants that are imputed in both UKB and TOPMed, but there are likely more population-specific variants imputed by TOPMed than by UKB, given the vast majority of individuals from the UKB panel are Europeans. Some discussions are warranted for a complete picture.

A practical issue is that some UKB individuals withdraw their consent. How would that affect the reference panel?

Reviewer #3 (Remarks to the Author):

The manuscript “A haplotype reference panel constructed from 490508 UK Biobank genomes improves genotype imputation for global populations” is well written and describes work that offers a valuable tool for imputing from the 500K UK Biobank reference set.

It is the first publicly available haplotype reference for the big UK Biobank set and the first imputation server against it as well. As such the manuscript merits publication.

I have only three comments, one major and two minor.

Major:

1. Throughout the manuscript there are many references to imputation quality using different names all involving r2: r2, imputation r2, imputation accuracy r2, imputation quality r2, imputation r2 score, imputation quality score r2, r2-score and r2-value.

The authors should choose a consistent terminology and preferably define the terminology they choose to use. There is considerable ambiguity in the terminology they use in the manuscript as it is and not altogether clear which measure they are referring to. I have seen similar terminology used in other papers so the problem may be more widespread.

On the one hand, we can have imputation r2, which is natural to interpret as the correlation squared between the imputed genotypes and the actual known genotypes (or alleles if phased alleles are known). The actual genotypes corresponding to the imputed ones for the 1000 Genomes data can be retrieved and used to calculate the actual imputation r2 for each variant. It is not clear in the manuscript if that is being done. From the terminology in Supplement 3 and Supplementary Figures 7-9 where they refer to r2-score for both the 1000G data and the Covid datasets, it seems they are not using the real genotypes for the 1000G in calculating imputation r2. If that is true, it should be remedied. It is very important to use real imputation r2 where possible for assessing performance. Since the Covid datasets only have GSA data, the real genotypes are not known and another measure has to be used.

Historically, imputation info was often used to refer to the variance ratio that is often used to estimate r2. If the r2-score they refer to is the same, it should be made clear. The paper nr 22 in the reference list derives the imputation info or score as an estimate of the imputation r2 using the assumption $E[X \mid Z]=Z$ where X is the actual allele and Z the imputed posterior probability, i.e. an unbiased imputation model. It is a fairly strong assumption and in assessing performance one wants to compare results without relying on such a strong assumption.

Minor:

2. The authors refer to their reference panel as the largest to date (to their knowledge). It is ok to qualify it as such as long as they say it is to their knowledge but maybe it would be better to call it the largest publicly available one since the 500K UK Biobank reference has been phased and used for imputation, although not available publicly.

3. In lines 62-65 the authors refer to their work on EagleImp for the 1000 Genomes Project dataset. They refer to the 1000G paper (ref 8) at the end of the sentence but I expected a reference to their EagleImp paper (ref 6). If they wanted to refer to 1000G and not their work there, maybe it should be made clear through parenthesis.

Reviewer #3 (Remarks on code availability):

I have not tested the code and only very briefly looked at it.
The eagleimp-web server has some faqs and howtos on its website for user to seek info/help.
There is also helpful material about its use in the Supplement with the manuscript.
eagleImp is described in another paper (ref 6) and has been in use. The code looks good.

The vcffilter has help on the github page with good hints about its use. The code itself is basic. An implementation using a library such as htslib would probably be more general. I must admit the use of a goto statement in the code is not pleasing to the eye but if it works it works.
The code is clear and restorevcf as well.

**Although we cannot publish your paper at this time, it may be appropriate for another journal in the Nature Portfolio. If you wish to explore other journals and transfer your manuscript, please use our manuscript transfer portal. You will not have to re-supply manuscript metadata and files, unless you wish to make modifications. For more information, please see our manuscript transfer FAQ page.

Version 1:

Decision Letter:

IMPORTANT: Please note the reference number: NG-LE68636R-Z Ellinghaus. This number must be quoted whenever you communicate with us regarding this paper.

22nd August 2025

Dear David,

Thank you for asking us to consider a resubmission of your revised manuscript entitled "A haplotype reference panel constructed from 490,508 UK Biobank genomes improves genotype imputation for global populations". I have discussed your appeal and supporting documentation with my editorial colleagues, and we invite you to proceed with a formal resubmission as proposed.

When preparing a revision, please ensure that it fully complies with our editorial requirements for format and style; details can be found in the Guide to Authors on our website (http://www.nature.com/ng/).

Please be sure that your manuscript is accompanied by a cover letter and separate point-by-point response detailing the changes you have made and your response to the points raised. At this stage, we will need you to upload:

1) A copy of the manuscript in MS Word .docx format.

2) The Reporting Summary: https://www.nature.com/documents/nr-reporting-summary.pdf

(Here you can read about the role of the Reporting Summary in reproducible science: https://www.nature.com/news/announcement-towards-greater-reproducibility-for-life-sciences-research-in-nature-1.22062)

Please use the link below to be taken directly to the site and view and revise your manuscript:

Link Redacted

With best wishes,
Kyle

Kyle Vogan, PhD
Senior Editor
Nature Genetics
https://orcid.org/0000-0001-9565-9665

Version 2:

Decision Letter:

4th November 2025

Dear David,

Your revised Letter "A haplotype reference panel constructed from 490,508 UK Biobank genomes improves genotype imputation for global populations" has been seen by two of the original reviewers. (Reviewer #1 was also asked to review the revision but was unable to provide a full review at this round.) You will see from the comments below that, while Reviewer #2 has no further requests, Reviewer #3 has highlighted a few additional points requiring clarification. We remain interested in the possibility of publishing your study in Nature Genetics, but we would like to consider your response to these points in the form of a further revision before we make a final decision on publication.

To guide the scope of the revisions, the editors discuss the referee reports in detail within the team, including with the chief editor, with a view to identifying key priorities that should be addressed in revision, and sometimes overruling referee requests that are deemed beyond the scope of the current study. In this case, we ask that you address all remaining technical points raised by Reviewer #3 with suitable revisions and clarifications.

In addition, although Reviewer #1 was unable to provide a full review at this round, this reviewer did express ongoing concerns about whether the imputation panel could remain online since UK Biobank's policy for using previously downloaded data sets states that the UKB-RAP must be used once the material transfer agreement expires or is renewed:

https://community.ukbiobank.ac.uk/hc/en-gb/articles/19968469678237-Can-I-still-use-the-data-that-I-have-previously-downloaded

We therefore ask that you clarify how the above policy impacts the long-term availability of the imputation server.

We hope that you will find this prioritized set of referee points to be useful when revising your study. Please do not hesitate to get in touch if you would like to discuss these issues further.

We therefore invite you to further revise your manuscript taking into account all reviewer and editor comments. Please highlight all changes in the manuscript text file. At this stage, we will need you to upload a copy of the manuscript in MS Word .docx or similar editable format.

We are committed to providing a fair and constructive peer-review process. Do not hesitate to contact us if there are specific requests from the reviewers that you believe are technically impossible or unlikely to yield a meaningful outcome.

When revising your manuscript:

*1) Include a “Response to referees” document detailing, point-by-point, how you addressed each referee comment. If no

action was taken to address a point, you must provide a compelling argument. This response will be sent back to the referees along with the revised manuscript.

*2) If you have not done so already, please begin to revise your manuscript so that it conforms to our Letter format instructions, available
here.
Refer also to any guidelines provided in this letter.

*3) Include a revised version of your Reporting Summary: https://www.nature.com/documents/nr-reporting-summary.pdf
It will be available to referees (and, potentially, statisticians) to aid in their evaluation if the manuscript goes back for peer review.
A revised checklist is essential for re-review of the paper.

Please be aware of our guidelines on digital image standards.

EXTENDED DATA FIGURES

When re-submitting your manuscript, please ensure that any supplementary figures and tables that are crucial to the manuscript's conclusions are converted into Extended Data figures and tables to increase visibility of these data. Extended Data figures and tables are online-only (present in the online PDF and full-text HTML versions of the paper), peer-reviewed display items that provide essential background to the article but are not included in the main article due to space constraints. A maximum of ten Extended Data display items (figures and tables) is permitted.

Please use the link below to submit your revised manuscript and related files:

Link Redacted

Note: This URL links to your confidential home page and associated information about manuscripts you may have submitted, or that you are reviewing for us. If you wish to forward this email to co-authors, please delete the link to your homepage.

We hope to receive your revised manuscript within 4-8 weeks. If you cannot send it within this time, please let us know.

Please do not hesitate to contact me if you have any questions or would like to discuss these revisions further.

Nature Genetics is committed to improving transparency in authorship. As part of our efforts in this direction, we are now requesting that all authors identified as 'corresponding author' on published papers create and link their Open Researcher and Contributor Identifier (ORCID) with their account on the Manuscript Tracking System (MTS), prior to acceptance. ORCID helps the scientific community achieve unambiguous attribution of all scholarly contributions. You can create and link your ORCID from the home page of the MTS by clicking on 'Modify my Springer Nature account'. For more information, please visit www.springernature.com/orcid.

We look forward to seeing the revised manuscript and thank you for the opportunity to review your work.

Sincerely,
Kyle

Kyle Vogan, PhD
Senior Editor
Nature Genetics
https://orcid.org/0000-0001-9565-9665

Referee expertise:

Referee #1: Genetics, statistical methods, imputation

Referee #2: Genetics, statistical methods, imputation

Referee #3: Genetics, statistical methods, imputation

Reviewers' Comments:

Reviewer #2 (Remarks to the Author):

The authors have carefully addressed my comments, particularly calculation of true R2 across all 26 sub-populations. I have no further comments.

Reviewer #3 (Remarks to the Author):

In their revision the authors have addressed most of the concerns made in the review of the first submission.

The terminology of the estimated R2 is handled well in the revision.

They have added a comparison between imputed data to real genotype data from 1000G project, lack of which was pointed out in the earlier review.

I have a few comments about the use and discussion of the added imputation quality metrics used in the current revision.

1. Regarding the use of SER for comparing phasing accuracy, I agree that it is a very good metric to assess phasing quality. The usual practice is to use trios to define the ground truth, remove parents, phase and compare the child's phase to the ground truth to calculate SER. In the paper, the authors use the 1000G haplotypes as ground truth. That is fine as long as they have reason to believe that the 1000G haplotype phase is much more reliable than the phase from their approach or from TOPMed. Otherwise, a better phasing would be penalized for being different.

2. It is fine to use MAE along with other quality metrics for comparison. It has its deficiencies though that must be kept in mind. Very rare variants have low MAE for obvious reasons since most will be imputed with the major allele. Even an imputation that imputes the major allele into everyone would have low MAE. The authors point out in lines 286-294 that imputation errors are smaller for low frequency variants and have smaller bias and then recommend the use of MAE in what follows. I find that logic weak.

3. The authors also use the correlation r2 as a quality metric. It is widely used and has many good properties. When errors occur in imputation of carriers for very rare variants, for instance, the r2 statistic captures that well while MAE does not. The authors begin by showing results for the superpopulations in 1KG but spend more time on discussing results for the 26 subpopulations, which all are very small (all with 85-108 samples except one which has 61 and another which has 113). It is true that it is tricky to assess r2 for rare variants in such small sample sets. It is fine to spend some time discussing it, but they should emphasize results for bigger sets where r2 is better estimated and the differences with the TOPMed imputation in that regard. When one has a bigger sample set, one can define bins based on target set frequencies and only use variants that are polymorphic in the target set. r2 is then well estimated.

4. Small target sets. One approach that they take for small target sets such as the 26 subpopulations is to combine markers within a bin to estimate one r2 value. That is one way to address the problem. Including many variants that may be nonpolymorphic for that subpopulation is detrimental for this analysis though. In the TOPMed paper, they classify their samples into 1KG superpopulations based on haplotype similarity with samples in 1KG and define variant classes based on frequency within the corresponding group in their data set. Something similar could be done here. I think it would be better than just use very low frequency variants based on the whole 500k UK Biobank set.

5. In lines 278-281, the authors comment that imputation is easier for low frequency variants than common ones if reference and study populations are poorly matched. The data from Supplementary Tables 10-11 and Figures 19-22 show that in all cases r2 increases with variant frequency and that more distant populations have lower r2 values. It might be argued that the difference is surprisingly small, but in any case, as it is their assertion seems strange.

Minor

6. The authors use mean values throughout their paper. I would consider a median to be a more robust estimator. In lines 202-208, they mention that knowing the average R2 in a category gives the user information about how many valid imputed variants can be expected. Some range metric like an interquantile range or for that matter how many variants have R2 above a threshold would be a much better metric to give that information.

7. I am assuming that all samples are imputed for all variants and that the comparison between their imputation and TOPMed are thus based on all the samples for all variants. Some imputation methods give missing values if they cannot impute. In such cases, a yield metric might be of value, and it is important in comparing two methods to base it on the same set of samples in each marker where both methods have an imputation for all selected samples.

8. In lines 486-7 in the Supplement, the authors specify what happens if they set the r2 values as 0 or 1 and then add “or completely ignoring...”. The last part of the sentence (quoted) probably belongs with the one before, not this one, at least not as it is written.

Version 3:

Decision Letter:

Our ref: NG-LE68636R2

9th July 2026

Dear David,

I apologize for the unusually long processing delay - thank you for your patience. Your revised manuscript "A haplotype reference panel constructed from 490,319 UK Biobank genomes improves genotype imputation for global populations" (NG-LE68636R2) has been seen by Reviewer #3. As you will see from the comments below, Reviewer #3 is satisfied with the revision pending a few remaining points, and therefore we will be happy in principle to publish your study in Nature Genetics as a Letter pending final revisions to address Reviewer #3's remaining points and to comply with our editorial and formatting guidelines.

We are now performing detailed checks on your paper, and we will send you a checklist detailing our editorial and formatting requirements soon. Please do not upload the final materials or make any revisions until you receive this additional information from us.

Thank you again for your interest in Nature Genetics. Please do not hesitate to contact me if you have any questions.

Sincerely,
Kyle

Kyle Vogan, PhD
Senior Editor
Nature Genetics
https://orcid.org/0000-0001-9565-9665

Reviewer #3 (Remarks to the Author):

Before continuing, I would like to emphasize that I believe the authors have done a good work and the paper is well written. I sincerely believe it merits publication. The remaining disagreements are focused on relatively minor things while the bulk of the work is very well done.

Although I pointed out a few things I would have liked to change, it is quite normal for scientists to disagree. The remaining comments below should be taken as minor complaints, most of which have to do with problems related to correlation r2 due to handling of variants non-polymorphic in the target set.

In my experience, the correlation r2 is the most reliable measure but I have had the luxury of working with either bigger target data sets and have defined variant bins based on target frequency or from a reference very similar to the target set. In such cases, correlation r2 is well defined and widely used as well (e.g. in the IMPUTE papers and in the TOPMed paper). Singletons or other such extremely rare variants must be handled differently, e.g. with pooled r2.

MAE can be used as a supplementary metric, although for very rare variants, it may not indicate well if the imputation of the minor allele is successful. One possible remedy would be to estimate error rate within carriers, but it is necessary to pool variants together to get a reliable estimate.

The numbers refer to my points in the earlier review which the authors answer in their response.

1.-2. I am happy with the authors' response.

3. The example the authors mention for YRI vs GBR in the rare variant bin shows that there are problems with the correlation r2 measure as it is being used by them, as they point out.

They seem to have put 0 for r2 when it cannot be computed. That obviously biases the mean towards 0. One plausible reason for the strange results they refer to is that, due to YRI being more polymorphic than GBR, there are fewer structural zeros in the r2 results for it.

The bottom line is that correlation r2 cannot be calculated for variants that are non-polymorphic in the target set. In that case, the pooled r2 result is much more reliable.

4. The authors have misunderstood me regarding binning variants. Of course variants must be binned for summary statistics. By “to combine markers within a bin to estimate one r2 value", I was referring to the pooled r2 method.

I understand the reason for their MAF-based binning using the UKB reference panel since they are working with such small target sample sizes. That means though that many variants within a bin may be non-polymorphic in the target population. I agree that the TOPMed strategy of defining MAF bins is probably not applicable for UK Biobank, but that does not make the problem go away. They have their reason to define their bins. The problem with the correlation r2 is related to this approach.

I believe putting 0/1 for non-polymorphic variants to calculate statistics is not good. It can work when it is very rare (e.g., when the reference is very similar to the target data). I would select the pooled estimate instead here.

Finally, I would like to point out that inflating variant bins with non-polymorphic variants will bias statistics such as MAE to make the results look better.

5. The sentence I referred to (599-601 in supplement) makes sense in relation to the example for YRI vs GBR in lines 586-590, but in between they refer to pooled correlation r2 results in Supplementary Tables 11-12 and Figures 19-22, which show a different scenario. I believe the sentence should come before referring to the pooled results. I for one went directly to the figures to see if the sentence made sense, since that was the last referred result before the sentence.

6. I agree that the mean is much more used than the median, although I believe the median is more reliable. It was just an observation on my behalf. Using the same metric as the majority of the field does is not a problem.

The (very) minor comment has to do with the statement that knowing the mean gives indication of how many variants will pass a threshold (lines 91-93). Knowing the mean gives an indication of overall quality but only a weak indication of how many pass a threshold. If the mean happens to be very close to the threshold, you can expect around 50% to pass. If on the other hand it is far away, you only know that the percentage passing will be somewhat/quite lower/higher than 50%.

7.-8. I am happy with the authors' reply.

I would like to finish by congratulating the authors on their good work and again emphasize that, even as it is, the paper merits publication.

INTERNAL USE ONLY AIP modules required: ["Clinical","Life Science"]

Open Access This Peer Review File is licensed under a Creative Commons Attribution 4.0 International License, which permits use, sharing, adaptation, distribution and reproduction in any medium or format, as long as you give appropriate credit to the original author(s) and the source, provide a link to the Creative Commons license, and indicate if changes were made.
In cases where reviewers are anonymous, credit should be given to 'Anonymous Referee' and the source.
The images or other third party material in this Peer Review File are included in the article’s Creative Commons license, unless indicated otherwise in a credit line to the material. If material is not included in the article’s Creative Commons license and your intended use is not permitted by statutory regulation or exceeds the permitted use, you will need to obtain permission directly from the copyright holder.
To view a copy of this license, visit https://creativecommons.org/licenses/by/4.0/

Point-by-point reply for NG-LE68636R1 "A haplotype reference panel constructed from 490,508 UK Biobank genomes improves genotype imputation for global populations"

Reviewer #1 (Remarks to the Author):
The authors present a haplotype reference panel constructed from 490,508 UK Biobank genomes that demonstrates improved imputation quality across diverse populations. However, my major concern relates to the legal and regulatory basis for constructing this reference panel. According to the authors, this research "has been conducted using the UK Biobank Resource under application number 139525." Based on publicly available information about this application (https://www.ukbiobank.ac.uk/enable-yourresearch/
approved-research/integrative-multi-omics-and-computational-phenotyping-for-precision-medicine-in-inflammatory-boweldisease), it is unclear whether the approved scope includes the construction of a reference panel hosted outside UK Biobank's secure infrastructure.
Furthermore, the authors employed an unconventional approach to data acquisition by downloading 151,561 unphased chunks of 20 kbp. This methodology appears to circumvent UK Biobank's standard data access protocols, as UK Biobank has implemented download limitations specifically to prevent bulk data extraction of this nature. Such an approach raises questions about compliance with approved data access terms and adherence to UK Biobank's data governance framework. Given these concerns, I believe it is essential for the authors to provide comprehensive documentation demonstrating that their approved application specifically covers reference panel construction and external hosting, along with evidence of any special permissions or exemptions obtained for their data extraction methodology (for example, see UK Biobank's data access exemption procedures: https://community.ukbiobank.ac.uk/hc/en-gb/articles/23418486279325-Applying-for-a-data-access-exemption). Without such documentation, the regulatory foundation for this otherwise scientifically valuable work remains questionable.

We thank Reviewer #1 for these important comments. The lay summary of our UK Biobank project #139525 on the UK Biobank website (https://www.ukbiobank.ac.uk/enable-your-research/approved-research/integrative-multi-omics-and-computational-phenotyping-for-precision-medicine-in-inflammatory-bowel-disease) was written on behalf of UK Biobank in very general terms for the public and refers only to "computational models" because the public usually has little knowledge of specific terms such as "imputation" or "haplotype reference panel". We therefore fully understand Reviewer #1's concerns as to whether we are authorized to create a UK Biobank imputation panel and offer it via our *EagleImp-Web* imputation service in Kiel, Germany.

Our work on the UK Biobank imputation reference panel and its accessibility via our *EagleImp-Web* imputation server was explicitly described in detail in the project description of our UK Biobank project #139525 (which Reviewer #1 unfortunately could not access via the UK Biobank website as it is not open to the public). We received permission from UK Biobank in project #139525 to build the UK Biobank imputation reference panel and offer it to the genetics community for the purpose of genotype imputation on our Kiel Imputation Server at the given URL https://hybridcomputing.ikmb.uni-kiel.de. We have compiled all contract documents and communications with UK Biobank and passed them to the editors of Nature Genetics (37 pages of documentation). We apologize to Reviewer #1 that we can only provide parts of the original documents related to the UK Biobank imputation reference panel here, as the full documents sent to Nature Genetics contain all our personal communication with UK Biobank, all project details as well as contract documents.

The work we have outlined in the manuscript to process the UK Biobank genotype data as part of our *EagleImp-Web* imputation service is as agreed in the project description no. 139525 and the associated Material Transfer Agreement (MTA). In accordance with the MTA, we have complied with all requirements regarding the downloading and processing of the UK Biobank data. This was explicitly reviewed and confirmed again by UK Biobank after submission of our annual report (email forwarded to Nature Genetics) in June 2025. In the annual report (also forwarded to Nature Genetics), we explicitly informed the UK Biobank team that we had been specifically asked by reviewers about the legal basis for our work and that our manuscript was under peer review at Nature Genetics. On July 25, 2025, UK Biobank again confirmed by email that we have met all requirements and that UK Biobank has no objections to publishing the UK Biobank imputation reference panels and hosting them via our *EagleImp-Web* imputation server, as it was described in our detailed project description of our UK Biobank project #139525.

The project description of our approved UK Biobank project #139525, which is unfortunately not publicly available, describes that we will create a UK Biobank imputation reference panel for genotype imputation and to offer its use via our secure webserver EagleImp-Web. The important sentence is the following: "Crucial for our PheWAS validation cohorts, we will create an UKB imputation reference panel using the UKB whole genome sequencing data for our secure and GDPR-compliant EagleImp-Web service (https://hybridcomputing.ikmb.uni-kiel.de; https://www.biorxiv.org/content/10.1101/2022.02.24.481790v1), specifically designed to construct large reference panels with millions of genomes, to enable miGut-Health members and the global genetics community to phase and impute GWAS datasets with UKB reference data (without giving them access to the UKB data) to accurately infer missing genotype data and expand the number of testable genetic variants."

Our project description was successfully confirmed by UK Biobank on October 24, 2023. We provided the Editors of Nature Genetics with screenshots from the AMS Portal. On October 26, 2023, the MTA contract and the project description were finally approved and signed by all parties on March 19, 2024, becoming effective on March 21, 2024.

The next question from Reviewer #1 was of course also legitimate, whether we have correctly analyzed the data according to the UK Biobank guidelines. Reference was made to the UK Biobank Community entry at https://community.ukbiobank.ac.uk/hc/en-gb/articles/23418486279325-Applying-for-a-data-access-exemption, as UK Biobank is currently in a transition phase from offline calculations to the use of a cloud-based UK Biobank Research Analysis Platform (UKB-RAP). However, this data access exemption does not apply to us, since we have an MTA with Annexes 1 and 2 from 2023 (which we handed over to the Editors of Nature Genetics), which allows us to download UK Biobank genotype data originated from sequence data to our own secure environment, in accordance with the MTA-based regulations, see also https://community.ukbiobank.ac.uk/hc/en-gb/articles/19968469678237-Can-I-still-use-the-data-that-I-have-previously-downloaded.

In our manuscript we described that we only extracted the genotypes and sample identifier from the huge amount of 151,561 available Graphtyper pVCF files using our tool vcffilter published on github (see manuscript), which is in line with Annex 1 of our MTA. The data download of genotypes and sampleIDs has been permitted for the project, with the exception that we do not download bulk whole genome sequence (WGS) and whole-exome sequence (WES) raw files such as FASTQ files or BAM/SAM files from the UK RAP system, see page Annex 1 in the MTA, sentence "excluding any WGS (whole genome sequence) or WES (whole exome sequence) files which must not be transmitted or downloaded from the research analysis platform". We would not have been able to download WGS or WES sequence data in the

order of 1.5 petabytes, nor would we have been willing to store the data locally. We only work with GraphTyper vcf genotype files pre-processed by UK Biobank, which do not contain any sequence information, but contain the finished genotypes of the genetic variants, which of course had to be finally processed on our secure servers for the creation and hosting of the UK Biobank Imputation Reference Panel.

#2 (Remarks to the Author):

In this manuscript, Wienbrandt et al presents a valuable resource, an imputation reference panel constructed from ~half million UK Biobank individuals. The manuscript is brief yet informative. The resource has high potential to benefit the large scientific community. There are many additional merits of the manuscript, including evaluation of the impact on downstream analyses such as association testing and fine-mapping, as well as the inclusion of chromosome X.

We thank Reviewer #2 for this positive feedback.

The biggest concern with the current analyses is that the evaluations are based on ESTIMATED imputation quality rather than TRUE imputation quality. Reference panels leading to better ESTIMATED imputation quality may simply reflect an over-estimation or mis-calibration rather than truly better quality. For example, a reference panel with low haplotype diversity due to smaller sample sizes or less heterogenous populations can result in unwarranted high confidence in imputation output. In addition, the estimated imputation quality metric has also been shown to deviate more from true quality for lower frequency variants, which larger reference panels such as the one presented here are most valuable. Therefore, the evidence provided in the current manuscript fails to support the value of the reference panel. More precisely, it is impossible to judge the value of the constructed reference panel with the results based on estimated imputation quality. The concern, however, can be rather easily addressed by masking some variants and evaluating the true quality.

We thank Reviewer #2 for this valuable feedback. We agree that we missed to compare our benchmark results with a known ground truth, as was done for example in the TOPMed reference panel publication (PMID 33568819, Methods section "Evaluation of imputation accuracy" and Supplementary Figure 37), where correlation r^2 benchmark results (with r^2 values averaged over variants) were calculated using 500 proxy individuals from the TOPMed reference panel assigned to the five superpopulations (AFR, AMR, EAS, EUR, SAS) of the 1000 Genomes Project.
Instead of working with proxy samples that resemble representatives from the superpopulations and the subpopulations of the 1000 Genomes Project, we instead compared the imputation results of our 1000 Genomes Project imputation benchmarks (based on 619,587 input variants available on Illumina's GSA taken for all 5 superpopulations and 26 subpopulations of the 1000 Genomes Project (see Supplementary Table 2)) with the ground truth genotypes of all variants available from the 1000 Genomes Project sequencing data, as we did for before for our estimated R^2 benchmarks. This new benchmark could only be conducted on a reduced number of variants shared between the UK Biobank reference panel, the TOPMed reference panel and the 1000 Genomes Project panel (30.1 million resp. 56.9 million, see new Figure 2).

To create a comprehensive overview of imputation quality based on the 1000 Genomes Project ground truth, we compared the results of three different phasing or imputation quality measures: Phasing switch

error rate (SER), mean absolute error (MAE) and correlation r^2 (for details on the calculation, see new sections 3.2.2 to 3.2.4 in the Supplementary Methods). We have included all new benchmark results in the new Results section, with new Supplementary Tables 5-11, Supplementary Figures 8-22 and Figure 2.

In summary, the additional benchmark results showed that the UK Biobank reference panel showed a reduction in SER compared to the widely used TOPMed r3 panel for all global superpopulations except the American superpopulation and for 25 of 26 subpopulations from the 1000 Genomes Project (Supplementary Figures 8-11, Supplementary Table 5), and a reduction in MAE imputation quality for four of five superpopulations and 12 subpopulations (8 subpopulations similar and 6 subpopulations better with the TOPMed panel) for variants with minor allele frequencies as low as 0.01% (Supplementary Figures 12-14, Supplementary Tables 6-7). Benchmark results based on correlation r^2 (with r^2 values averaged across variants) showed correlation r^2 for both UK Biobank reference panels and the TOPMed r3 panel (Supplementary Table 8 and 9, upper panel; Supplementary Figures 15 and 16) consistent with the previous correlation r^2 benchmarks (also r^2 values averaged across variants) based on the 500 proxy individuals from the TOPMed panel (PMID 33568819).

However, because no subpopulations with smaller sample sizes were examined in the TOPMed publication (PMID 33568819), we additionally calculated correlated r^2 for the 26 subpopulations (see Supplementary Figures 15-22; Supplementary Tables 8-11) and found that correlated r^2 shows problems especially for low minor allele frequencies (MAFs); this applies both to the UK Biobank as well as the TOPMed reference panel), similar to what was observed for the estimated R^2 measure and reported by Das et al. 2018 (PMID 29799802).

Correlation r^2 is usually computed as the squared Pearson correlation coefficient. This measure has a strong weakness when it comes to observations or expectations with low or without variance, as it measures the relation of covariance to uncorrelated variance. Without any variance in the observed or expected data, the calculation term simply contains a 0 in the denominator and can therefore not be calculated. A workaround solution is to set r^2 to zero in those cases, which is not correct as completely correctly imputed variants without variance (i.e. e.g. all genotypes are 0) drop the average r^2 (computed over all variants) instead of increasing it.

We observed in our benchmarks that the latter is very often the case, especially for the European superpopulation benchmark dataset, where far more variants were correctly imputed with 0-genotypes using our UKB reference panel than the TOPMed panel. This effect was even stronger for the European subpopulations as the sample size was smaller and for lower MAF categories, where the probability to have a genotype imputed as 1 or 2 instead of 0 was much lower, implying even less variance per variant. Further, low variances also imply a low denominator in those cases a correlation r^2 can theoretically be computed, but will very likely lead to a strong numerical error. For example, we observed a variant-wise average correlation r^2 for the African superpopulation (AFR) of around 0.5 for the low MAF range category between 0.0001 and 0.001 (for UKB as well as for TOPMed) while the European superpopulation (EUR) only results in an r^2 of approx. 0.15 in this category (again for UKB and TOPMed). Further, the subpopulation ACB (sub of AFR) showed r^2=0.4 in this category while GBR (Great Britain, subpopulation of EUR) only showed an r^2=0.04 (again UKB and TOPMed differ only slightly). The interpretation of such results definitely leads to the wrong conclusion that both UKB and TOPMed panel imputation were better suited to impute African study populations than European ones,

which is not the case (from a population genetics point of view and supported by figures using the MAE measure).

In an attempt to compensate for this weakness of averaging correlation r^2 values based on such small amounts of datapoints with r^2 values averaged over variants, we calculated correlation r^2 based on all datapoints of an rpMAF range category. In detail, we used all datapoints (i.e. imputed haplotype dosages in comparison to the expected haplotypes from the 1000 Genomes reference ground truth)) over all samples and all variants from an rpMAF range category, but separately for each chromosome file. For the final correlation r^2, we averaged the values for each chromosome file weighted by the number of variants. We also present these results in our Supplementary Material and include a paragraph for discussion in our manuscript. In brief, we now observed a much higher correlation especially for low MAF categories as we removed the impact of $r^2=0$ values for perfectly imputed variants. Also, numeric errors from small denominators were reduced by taking a much higher set of datapoints into account. Now, the results correlate much better with our observations from measuring the MAE. However, correlation r^2 values of more than 0.9 for the 0.0001-0.001 MAF category seem very unlikely, although now calculated on a much more reliable basis.

We have summarized our results in lines 255-289 in the manuscript as follows:

"However, the correlation values r^2 calculated separately for the 26 subpopulations (Supplementary Tables 8 and 9, middle panel; Supplementary Figures 17 and 18) showed a weakness of the correlation r^2 measure for imputation quality for low allele frequencies, leading to potentially biased results due to small sample sizes especially for rpMAF range categories [0.1%, 1%) and [0.01%, 0.1%) and because in cases with small or no variance the correlation r^2 simply cannot be calculated due to a denominator of zero; thus, setting the correlation r^2 to 0 for perfectly imputed genotypes for all benchmark samples leads to (disproportionately) lower average correlation r^2 values (see details and formulas for correlation r^2 in Supplementary Section 3.2.4). From a population genetics perspective, for example, it is also unlikely that the imputation quality at [0.01%, 0.1%) for the rpMAF-filtered UKB reference panel (or TOPMed panel) for GBR is worse than for YRI, with a correlation r^2 value of 0.04 (0.04) for GBR compared to YRI 0.40 (0.44) (Supplementary Tables 8 and 9, middle panel; Supplementary Figures 17 and 18), knowing that 93.82% of UK Biobank participants reported their ancestry as "White British" and only 1.56% of participants reported their ancestry as "Black" or "Black British" (Supplementary Table 1).
In an attempt to provide enough data points to calculate correlation r^2 for the 26 subpopulations individually, we calculated correlation r^2 for all three imputation reference panels additionally based on the total number of observations across all samples and variants per rpMAF range category (instead of correlation r^2 averaged across variants) showing high correlation values r^2 for all 26 subpopulations (Supplementary Tables 10-11; Supplementary Figures 19-22), regardless of whether the genetic similarity between our three reference panels and the target (input) subpopulation benchmark dataset was high or low.
These results from correlation r^2 would imply that the imputation of genotypes appears to be much easier for low-frequency variants than for common variants in the case that the study population and individuals from the reference panel are poorly matched. However, since the algorithm most frequently estimates the more frequent allele for low rpMAF categories purely by chance and thus correlation variance is usually low just by chance, this often leads to a biased correlation r^2 for low range rpMAF categories (and a false and underestimated correlation r^2 set to 0 for perfectly imputed genotypes, see text above).

Note that smaller imputation errors for low-frequency variants than for common variants were also observed in calculations based on MAE (Figure 2; Supplementary Figures 12-14), but with a smaller bias because the difference between the estimate and the ground truth was not measured on the basis of variances."

We have further summarized a detailed explanation of the advantages and disadvantages of all imputation quality metrics in the new sections 3.2.1, 3.2.3. and 3.2.4 in the form of text and formulas. Based on the observations made here, which are directly based on the ground truth data from the 1000 Genomes Project, we now make the following recommendation (lines 289-294):

"For this reason, we recommend the measure of imputation quality MAE (which is not based on correlation) for benchmark analyses with small ground truth datasets such as the 1000 Genomes Project data, and in general the measure SER for benchmarks of phasing quality, as well as the estimated R^2 (available for all variants of a reference panel) for post-imputation filtering of poorly imputed variants from GWAS input datasets."

A few more specific comments are listed below.

L145-147: instead of using cumulative categories, disjoint MAF categories (i.e., 0.01-0.1%, 0.1-1%, >= 1%) are recommended to avoid inflated imputation quality estimate by including common variants (which are well imputed even with earlier reference panels such as 1KG).

We thank you for this suggestion. For the new benchmarks based on mean absolute error (MAE) and correlation r^2 (line 216), we have implemented this suggestion and present these results based on the ground truth results at four rpMAF range categories ([0.01%, 0.1%), [0.1%, 1%), [1%, 10%), [10%, 50%]), see answer above.
We apologize for an incorrect mapping of our correctly measured data to the three MAF categories we used to present the results in the world map (Figure 1). We corrected Figure 1 accordingly. However, for the estimated R^2 results (line 157-198) we still suggest presenting cumulative values for the following reasons (see justification added to line 199):
"Imputation quality based on estimated R^2 is a useful information for imputation users as it is available for each variant from the reference panel (here up to 361 million variants) and is usually the only available measure for the exclusion of poorly imputed variants after phasing and imputation of GWAS input datasets. Knowing the average estimated R^2 for a given MAF category (cumulative counts shown in Figure 1 and Supplementary Figures 6 and 7) gives the user an approximate information on how many valid imputed variants can be expected from the imputation depending on the study population and the chosen minimum R^2 filter, which removes unreliable variants below the R^2 threshold from the imputation output (see EagleImp-Web features in Table 2)."
With the cumulative count, the figures also remain roughly comparable with the figures from previous publications on the TOPMed (PMID 33568819) and the Genomics England (GEL) (PMID 39134668) imputation panel, where a cumulative calculation was also carried out.

In the manuscript, the authors only compared variants that are imputed in both UKB and TOPMed, but there are likely more population-specific variants imputed by TOPMed than by UKB, given the vast majority of individuals from the UKB panel are Europeans. Some discussions are warranted for a complete picture.

We thank Reviewer #2 for this valuable comment. We added the following sentence in line 399 to the discussion:
"We would like to point out that the TOPMed r3 panel with a total of 244,838,777 panel-specific variants has more panel-specific variants than the full UK Biobank panel with 161,298,331 panel-specific variants, which makes it possible to impute more study population-specific variants from non-European populations with the TOPMed panel."

A practical issue is that some UKB individuals withdraw their consent. How would that affect the reference panel?

If participants withdraw their consent from UK Biobank, the genotypes of these participants should not be used in future analyses. The benchmarks presented here are based on the most recent version of our UKB reference panel and the participant removal list sent by UK Biobank, dated December 17, 2024. On August 18, 2025, UK Biobank sent us an updated list of participants to exclude from future analyses in our UK Biobank project 139525; however, analyses that have already been completed do not need to be repeated. For the current UKB reference panels, we refer to the participant removal list from December 17, 2024, as our analyses are complete. The current list of participants who have withdrawn will be considered in our upcoming release of the reference panel, which we decided to update once a year. Although this is not contractually required, and to our knowledge, is not done by other imputation servers, we believe that we should comply with the request of UK Biobank study participants to withdraw their consent, as their reference data is used in every phasing and imputation on our imputation server.

Removing participants from existing reference panels requires considerable amount of computation, as the reference panel files must be unpacked, the participant data removed, and the remaining data repacked. Since number of participants who withdraw their consent is generally low (111 participants as of December 17, 2024), we do not expect any significant negative impact on the quality of our imputation panels.

Reviewer #3 (Remarks to the Author):

The manuscript "A haplotype reference panel constructed from 490508 UK Biobank genomes improves genotype imputation for global populations" is well written and describes work that offers a valuable tool for imputing from the 500K UK Biobank reference set.

It is the first publicly available haplotype reference for the big UK Biobank set and the first imputation server against it as well. As such the manuscript merits publication.

We thank Reviewer #3 for this positive feedback.

I have only three comments, one major and two minor.

Major:

1. Throughout the manuscript there are many references to imputation quality using different names all

involving r2: r2, imputation r2, imputation accuracy r2, imputation quality r2, imputation r2 score, imputation quality score r2, r2-score and r2-value.

We would like to thank the reviewer for pointing this out. We apologize that we sometimes used different terms for the same thing. We have now corrected this and use "R^2" (note the capital "R") for estimated R^2 together with the term "estimated" and small "r^2" for the true correlation r^2 (squared PCC) together with the term "correlation". This should now make it clear to the reader which R^2/r^2 we are referring to. See also our new detailed explanations and formulas in Section 3.3 in the Supplementary Material.

The authors should choose a consistent terminology and preferably define the terminology they choose to use. There is considerable ambiguity in the terminology they use in the manuscript as it is and not altogether clear which measure they are referring to. I have seen similar terminology used in other papers so the problem may be more widespread.

On the one hand, we can have imputation r2, which is natural to interpret as the correlation squared between the imputed genotypes and the actual known genotypes (or alleles if phased alleles are known). The actual genotypes corresponding to the imputed ones for the 1000 Genomes data can be retrieved and used to calculate the actual imputation r2 for each variant. It is not clear in the manuscript if that is being done. From the terminology in Supplement 3 and Supplementary Figures 7-9 where they refer to r2-score for both the 1000G data and the Covid datasets, it seems they are not using the real genotypes for the 1000G in calculating imputation r2. If that is true, it should be remedied. It is very important to use real imputation r2 where possible for assessing performance. Since the Covid datasets only have GSA data, the real genotypes are not known and another measure has to be used.

Historically, imputation info was often used to refer to the variance ratio that is often used to estimate r2. If the r2-score they refer to is the same, it should be made clear. The paper nr 22 in the reference list derives the imputation info or score as an estimate of the imputation r2 using the assumption $E[X | Z]=Z$ where X is the actual allele and Z the imputed posterior probability, i.e. an unbiased imputation model. It is a fairly strong assumption and in assessing performance one wants to compare results without relying on such a strong assumption.

We thank Reviewer #3 again for pointing out the importance of the different r^2 measures. We now gave a very detailed response regarding this topic to Reviewer #2, as it was a similar issue. In summary, we included benchmark results for true correlation r^2 including a detailed discussion in our manuscript, including all formulas and further explanations in the new sections 3.2.1 (estimated imputation R^2), 3.2.2 (Switch Error Rate), 3.2.3 (Mean Absolute Error), and 3.2.4 (true correlation r^2). In the detailed response to Reviewer #2, we also pointed out serious problems with correlation r^2 computations for small samples sizes and low allele frequencies, and recommended instead using the measures mean absolute error (MAE) and switch error rate (SER) (which we also calculated in our new version of the manuscript based on the 1000 Genomes Project sequence data), if the ground-truth is known. Otherwise, if the ground-truth is not known, which is usually the case when imputing a study population, one must rely on a measure which is based purely on the imputation results. As Reviewer #3 correctly pointed out, Das et al. (2018) presented a measure for estimated R^2 which is fairly based on the mentioned strong assumption that $E[X|Z]=Z$, i.e. a perfectly calibrated panel where the expected values from the panel given the imputed dosages (posterior haplotype probabilities) are directly reflected by those dosages. However, although this assumption probably does not hold especially for different populations, it is

commonly used by recent imputation tools, including our tool EagleImp and minimac4 (which is used in the TOPMed service).
As all imputation quality measures shown in our manuscript have advantages and disadvantages and we discuss these, we prefer to still show results for estimation R^2 (in addition to switch error rate, mean absolute error and true correlation r^2) because the newly introduced measures only give an indication of the true imputation quality, as they are only based on a small fraction of all imputed variants (namely the variants shared between our UKB panels, the TOPMed panel and the 1000 Genomes reference panel, which account less than 16% of the complete UKB reference panel).

Minor:

2. The authors refer to their reference panel as the largest to date (to their knowledge). It is ok to qualify it as such as long as they say it is to their knowledge but maybe it would be better to call it the largest publicly available one since the 500K UK Biobank reference has been phased and used for imputation, although not available publicly.

We agree with Reviewer #3 to call it the "to our knowledge largest whole-genome haplotype reference panel publicly available for imputation to date". We modified the corresponding phrases in the manuscript. However, we are not aware that an imputation reference panel has been constructed from the nearly 500,000 UK Biobank participants to date, nor that this has been used for imputation purposes for any kind of GWAS data. For this reason, we would respectfully refrain from making this claim. The only publication on the construction of a UK Biobank imputation panel (however, not publicly available) that we found used an earlier version of the UK Biobank WGS data with 150,119 participants (Hofmeister et al., Nat.Gen., 2023, PMID 37386248), which we cited for the introduction of the SHAPEIT5 software in our manuscript.

3. In lines 62-65 the authors refer to their work on EagleImp for the 1000 Genomes Project dataset. They refer to the 1000G paper (ref 8) at the end of the sentence but I expected a reference to their EagleImp paper (ref 6). If they wanted to refer to 1000G and not their work there, maybe it should be made clear through parenthesis.

We thank the reviewer for pointing out that this might be unclear. We have restructured the sentence to clarify the references to our work and the 1000 Genomes publication as follows: "we previously showed that for input datasets from diverse human populations of the 1000 Genomes Project [8] EagleImp provides at least equal or better phasing and imputation quality than Eagle2 and PBWT in terms of phasing switch error rate and imputation genotype error rate [6]."

Reviewer #3 (Remarks on code availability):

I have not tested the code and only very briefly looked at it.
The eagleimp-web server has some faqs and howtos on its website for user to seek info/help.
There is also helpful material about its use in the Supplement with the manuscript.
eagleImp is described in another paper (ref 6) and has been in use. The code looks good.

We appreciate the positive feedback on our software code.

The vcffilter has help on the github page with good hints about its use. The code itself is basic. An

implementation using a library such as htslib would probably be more general. I must admit the use of a goto statement in the code is not pleasing to the eye but if it works it works. The code is clear and restorevcf as well.

We thank Reviewer #3 again for the feedback. We agree that the code is “basic”, and we intentionally did not want to use the htslib library (as we do in our EagleImp software) as htslib is more flexible on the one hand, but also uncomfortably slow on the other. Since the format of the VCF files in the UKB RAP is fixed and unique, we could directly target this structure to process the files in a very efficient way, especially to reduce the processing time on the UKB RAP and thus the cost (our first version with htslib was up to three times slower). We agree the use of goto statements in native C code is not always pretty to look at, but not uncommon.

Point-by-point reply for NG-LE68636R2 "A haplotype reference panel constructed from 490,319 UK Biobank genomes improves genotype imputation for global populations"

Response to the Editors and Reviewers:

We thank the editors and reviewers for their careful evaluation of our revised manuscript and for the constructive comments. We have addressed all remaining points raised by Reviewer #3 and clarified the issue raised by Reviewer #1 regarding long-term availability of UKB-based imputation. Below we provide detailed, point-by-point responses.

Additional adjustments:

We updated the reported UKB reference panel size to 490,319 individuals after removing 189 participants who had withdrawn consent in the meantime. Because these removals represent less than 0.04 % of the cohort, they do not materially affect allele frequencies or haplotype structure.

In parallel, we invested additional development time to expand the resource: both UKB reference panels (UKB complete and UKB MAF0.0001) are now available for genome builds hg19 and hg38. All four panels are listed in Table 1. No methodological changes were introduced.

As requested, the manuscript has been edited to meet the *Nature Genetics Letter* format requirements, including text length, structure, and display item limits. We have therefore moved many text blocks to the Supplementary Information.

Reviewer #1 (via Editor):

The reviewer expressed ongoing concerns about whether the imputation panel could remain online, given that UK Biobank's policy for using previously downloaded data sets states that the UKB-RAP must be used once the material transfer agreement expires or is renewed.

UK Biobank's current data-access policy specifies that analyses involving UKB genotype data must be executed within the UK Biobank Research Analysis Platform (UKB-RAP) once the Material Transfer Agreement reaches the renewal stage. To ensure long-term reproducibility in full alignment with this policy, we have now implemented a cloud-executing version of *EagleImp* (*EagleImp-RAP*) that runs natively within UKB-RAP.

All UKB-based benchmark analyses reported in the manuscript can now be reproduced directly within UKB-RAP, independent of the secure standalone compute environment used for the initial benchmark evaluations. No methodological changes were introduced; the addition of *EagleImp-RAP* ensures that reproducibility and long-term availability fully conform to current UKB data-governance requirements.

We have clarified this in the revised manuscript (Introduction, Results, Online Methods, Data Availability).

Referee expertise:
Referee #1: Genetics, statistical methods, imputation
Referee #2: Genetics, statistical methods, imputation
Referee #3: Genetics, statistical methods, imputation

Reviewers' Comments:

Reviewer #2 (Remarks to the Author):

The authors have carefully addressed my comments, particularly calculation of true R^2 across all 26 sub-populations. I have no further comments.

We thank the reviewer for the positive assessment.

Reviewer #3 (Remarks to the Author):

In their revision the authors have addressed most of the concerns made in the review of the first submission.

The terminology of the estimated R^2 is handled well in the revision.

They have added a comparison between imputed data to real genotype data from 1000G project, lack of which was pointed out in the earlier review.
I have a few comments about the use and discussion of the added imputation quality metrics used in the current revision.

1. Regarding the use of SER for comparing phasing accuracy, I agree that it is a very good metric to assess phasing quality. The usual practice is to use trios to define the ground truth, remove parents, phase and compare the child's phase to the ground truth to calculate SER. In the paper, the authors use the 1000G haplotypes as ground truth. That is fine as long as they have reason to believe that the 1000G haplotype phase is much more reliable than the phase from their approach or from TOPMed. Otherwise, a better phasing would be penalized for being different.

We appreciate this point and now clarify that haplotypes from Phase 3 of the 1000 Genomes Project (PMID 26432245), generated using a family-based scaffold, are widely used as ground-truth haplotypes in phasing evaluations (e.g., PMID 36055201) and in re-phasing studies that explicitly maintain consistency with this established scaffold (e.g., PMID 34478634). Because these haplotypes incorporate pedigree-based phase information and have been extensively validated across multiple populations, they are considered a high-quality reference for SER benchmarking. This clarification has been added to the Results and Supplementary Methods (Section 4.2.2).

2. It is fine to use MAE along with other quality metrics for comparison. It has its deficiencies though that must be kept in mind. Very rare variants have low MAE for obvious reasons since most will be imputed with the major allele. Even an imputation that imputes the major allele into everyone would have low MAE. The authors point out in lines 286-294 that imputation errors are smaller for low frequency variants and have smaller bias and then recommend the use of MAE in what follows. I find that logic weak.

We agree that MAE has known limitations for low-frequency variants, since imputing the major allele can yield artificially low errors. This limitation was already articulated in the previous manuscript: smaller imputation errors for rare variants were observed when using MAE (previous lines 286-294; Figure 2; Supplementary Figs. 12-14), "but with a smaller bias because the difference between estimate and ground truth was not measured on the basis of variances" — unlike correlation-based metrics such as r^2.

Our intention was not to recommend MAE as a standalone quality metric, but to explain why MAE remains informative when benchmarking very small ground-truth datasets such as 1000G subpopulations, where both estimated R^2 and empirical r^2 become unstable for rare variants. MAE quantifies absolute dosage deviation without variance-based normalization and thus provides complementary information under such conditions.

To make this role clearer, the text has been revised to insert "…as a secondary metric…" into the new Supplementary Section 5. For real-world GWAS applications, we continue to recommend estimated R^2 for post-imputation filtering and SER for phasing accuracy.

3. The authors also use the correlation r2 as a quality metric. It is widely used and has many good properties. When errors occur in imputation of carriers for very rare variants, for instance, the r2 statistic captures that well while MAE does not. The authors begin by showing results for the superpopulations in 1KG but spend more time on discussing results for the 26 subpopulations, which all are very small (all with 85-108 samples except one which has 61 and another which has 113). It is true that it is tricky to assess r2 for rare variants in such small sample sets. It is fine to spend some time discussing it, but they should emphasize results for bigger sets where r2 is better estimated and the differences with the TOPMed imputation in that regard. When one has a bigger sample set, one can define bins based on target set frequencies and only use variants that are polymorphic in the target set. r2 is then well estimated.

We agree that estimates of correlation r^2 become unstable in very small target sets such as the 26 subpopulations of the 1000 Genomes Project, particularly for low-frequency variants. While correlation r^2 can capture certain types of imputation errors for rare variants, this does not generalize across all settings because both MAE and correlation r^2 exhibit distinct bias structures, as outlined in our response to point 2. In particular, correlation-based metrics depend heavily on variance normalization, whereas MAE reflects absolute dosage deviation; neither metric provides uniformly unbiased estimates for very rare variants in small cohorts.

As stated in the manuscript, variant-wise r^2 can produce misleading patterns when allele frequencies between reference and target sets differ substantially. For example (previous lines 264-271), at rpMAF 0.01-0.1 %, r^2 for GBR is 0.04 (0.04) for the UKB rpMAF-filtered panel (and similarly for TOPMed), whereas YRI shows $r^2 = 0.40$ (0.44), despite the underlying demographic composition of UKB (93.85 % White British vs. 1.56 % Black/Black British; Supplementary Table 2). From a population-genetics perspective, it is implausible that imputation accuracy would be genuinely worse for GBR than YRI; this reflects metric instability rather than algorithmic performance.

We also agree that more robust conclusions arise from larger superpopulations. However, the absolute value of empirical r^2 depends strongly on the estimation approach: variant-wise r^2 (Supplementary Fig. 15) and pooled r^2 (Supplementary Fig. 19) can invert the relative ranking of EagleImp and TOPMed: Using variant-wise r^2, TOPMed often appears slightly ahead. Using pooled r^2, EagleImp often appears ahead. Because both results arise from the same imputed dosages, this inversion demonstrates that direct quantitative comparison of absolute r^2 values between EagleImp and TOPMed is inherently method-dependent. Because of these statistical limitations, we do not interpret small numerical differences in correlation r^2 (Supplementary Figures 15 and 19) as meaningful indicators of relative performance between the two reference panels. We have clarified this point in the manuscript (lines 121-126 and Supplementary Section 5).

4. Small target sets. One approach that they take for small target sets such as the 26 subpopulations is to combine markers within a bin to estimate one r2 value. That is one way to address the problem. Including many variants that may be nonpolymorphic for that subpopulation is detrimental for this analysis though. In the TOPMed paper, they classify their samples into 1KG superpopulations based on haplotype similarity with samples in 1KG and define variant classes based on frequency within the corresponding group in their data set. Something similar could be done here. I think it would be better than just use very low frequency variants based on the whole 500k UK Biobank set.

We agree that using UKB-wide allele frequencies (rpMAF) can yield bins containing variants that are monomorphic or nearly monomorphic in some 1000G subpopulations, which often leads to unstable r^2 values - including undefined or zero-valued estimates where variance is absent.

However, we emphasize that allele-frequency binning is not one of several possible approaches but a methodological requirement for interpreting SER, MAE, variant-wise r^2, pooled r^2, and estimated R^2. Without binning, these metrics are not interpretable: SER cannot be defined per variant; correlation r^2 is frequently undefined or numerically unstable due to zero variance even in larger cohorts. For these reasons, all established imputation benchmarking frameworks - including TOPMed - group variants into MAF bins before computing accuracy metrics.

These issues are amplified in the small 1000G subpopulations (61-113 individuals), where monomorphic variants are common and r^2 becomes dominated by variance-driven artefacts rather than true imputation accuracy (see responses to points 2 and 3). Consequently, stable MAF-based binning is essential for minimizing statistical artefacts for both EagleImp and TOPMed.

Our rationale for MAF-based binning using the UKB reference panel MAF (rpMAF) is that it provides far more stable frequency estimates than population-specific MAFs from 1000G subpopulations, whose sample sizes are too small for reliable stratification, particularly for variants below 1 %. Using 1000G-specific MAFs would result in severe frequency misclassification and reduce interpretability.

Regarding the reviewer's suggestion to follow the TOPMed strategy (assigning samples to superpopulations by haplotype similarity and defining MAF bins within those groups), we respectfully note that this is not applicable for UKB. The UKB reference panel is overwhelmingly homogeneous (93.85 % White British; Supplementary Table 2), and the very small numbers of non-British ancestry samples do not permit robust haplotype-based clustering into 1000G-like superpopulations. Any MAF bins derived from such inferred subgroups would be dominated by statistical noise and would not correspond to the 1000G subpopulations used for benchmarking.

For these reasons, the TOPMed-style procedure cannot be meaningfully reproduced for UKB, and UKB-wide MAF remains the only stable and interpretable basis for allele-frequency binning in this context (also since per-variant rpMAFs from the TOPMed r3 panel are not publicly available).

5. In lines 278-281, the authors comment that imputation is easier for low frequency variants than common ones if reference and study populations are poorly matched. The data from Supplementary Tables 10-11 and Figures 19-22 show that in all cases r2 increases with variant frequency and that more distant populations have lower r2 values. It might be argued that the difference is surprisingly small, but in any case, as it is their assertion seems strange.

We respectfully disagree with the reviewer's interpretation. The sentence referred to by the reviewer "These results from correlation r^2 would imply that the imputation of genotypes appears to be much easier for low-frequency variants than for common variants in the case that the study population and individuals from the reference panel are poorly matched." (previous lines 278-281) does not assert that low-frequency variants are inherently easier to impute. Rather, the passage described a statistical artefact of correlation r^2 in settings with population mismatch: when true minor allele frequencies are extremely low, dosage vectors lack variance, causing r^2 to inflate or collapse to zero even when imputation is perfect. This is explicitly stated in the manuscript and reflects the behaviour of the metric, not the algorithm. Since the text (now part of Supplementary Section 5) already makes this clear, no changes were necessary.

Minor

6. The authors use mean values throughout their paper. I would consider a median to be a more robust estimator. In lines 202-208, they mention that knowing the average R2 in a category gives the user information about how many valid imputed variants can be expected. Some range metric like an interquantile range or for that matter how many variants have R2 above a threshold would be a much better metric to give that information.

We appreciate the reviewer's comment regarding the use of mean versus distributional summaries. While median or interquartile ranges are robust descriptors, our use of mean estimated R^2 follows established conventions in imputation benchmarking, including the TOPMed study (PMID: 33568819), where mean (average) r^2 is the primary metric.

Our intention in previous lines 202-208 was not to suggest that the mean is a superior estimator, but to explain why the mean estimated R^2, as reported by all major imputation servers and software tools, is directly interpretable for downstream GWAS filtering. Because estimated R^2 is a per-variant quality measure, the average estimated R^2 within a MAF bin provides a practical indication of how many variants are likely to exceed a chosen R^2 filter (e.g. $R^2 > 0.3$ or > 0.8). This is why EagleImp - like other imputation frameworks - relies on per-variant estimated R^2 rather than distributional summaries for variant-level filtering.

To address the reviewer's suggestion, we have revised the text at new lines 89-94. We believe this clarification addresses the reviewer's concern while maintaining consistency with field-wide reporting standards.

7. I am assuming that all samples are imputed for all variants and that the comparison between their imputation and TOPMed are thus based on all the samples for all variants. Some imputation methods give missing values if they cannot impute. In such cases, a yield
metric might be of value, and it is important in comparing two methods to base it on the same set of samples in each marker where both methods have an imputation for all selected samples.

All comparisons between EagleImp (using the UKB reference panels) and the TOPMed imputation Server (using the TOPMed r3 panel) were performed on exactly the same set of variants and samples. For SER, MAE, and r^2, only variants present in UKB, TOPMed and 1000G were included (see Supplementary Fig. 5).

Both EagleImp and the TOPMed imputation server generate an imputation output containing dosage values for every variant present in the reference panel. Thus, neither method produced missing imputed values in our benchmark analyses. Because no missingness occurred and all accuracy metrics were computed on matched sample-by-variant matrices, a yield metric would not provide additional information. We now note this explicitly in the Supplementary Section 4.2.

8. In lines 486-7 in the Supplement, the authors specify what happens if they set the r2 values as 0 or 1 and then add "or completely ignoring...". The last part of the sentence (quoted) probably belongs with the one before, not this one, at least not as it is written.

The reviewer is correct that the wording in the original Supplement combined unrelated ideas into a single sentence. The sentence in the Supplementary Methods unintentionally combined two unrelated statements: (i) the behaviour of correlation r^2 when variance is zero, and (ii) exploratory alternatives we had considered but not used (setting $r^2 = 1$ or excluding such variants). To avoid confusion, we have removed the phrase "as well as setting correlation $r^2 = 1$ or completely ignoring these values in the calculation of the average".

The revised text in Supplementary Section 4.2.4 now reads: "Especially for variants with very low MAF we lost a significant proportion of r^2 values (especially for UKB imputations), even when such variants were perfectly imputed with no errors but exhibited no variation in the target samples (e.g. MAF = 0). In these cases, the denominator of the correlation r^2 formula is zero, leading to an undefined r^2 which we set to zero to be able to calculate a consistent average."

No alternative imputation or r^2-handling strategies are discussed anymore, as suggested by the reviewer.

Point-by-point reply for NG-LE68636R2 "A haplotype reference panel constructed from 490,319 UK Biobank genomes improves genotype imputation for global populations"

Reviewer #3
(Remarks to the Author):

Before continuing, I would like to emphasize that I believe the authors have done a good work and the paper is well written. I sincerely believe it merits publication. The remaining disagreements are focused on relatively minor things while the bulk of the work is very well done.

We sincerely thank the reviewer for their positive assessment.

Although I pointed out a few things I would have liked to change, it is quite normal for scientists to disagree. The remaining comments below should be taken as minor complaints, most of which have to do with problems related to correlation r2 due to handling of variants non-polymorphic in the target set.

In my experience, the correlation r2 is the most reliable measure but I have had the luxury of working with either bigger target data sets and have defined variant bins based on target frequency or from a reference very similar to the target set. In such cases, correlation r2 is well defined and widely used as well (e.g. in the IMPUTE papers and in the TOPMed paper). Singletons or other such extremely rare variants must be handled differently, e.g. with pooled r2.

MAE can be used as a supplementary metric, although for very rare variants, it may not indicate well if the imputation of the minor allele is successful. One possible remedy would be to estimate error rate within carriers, but it is necessary to pool variants together to get a reliable estimate.

We agree with the reviewer that measuring imputation accuracy for very low MAF variants is challenging, especially when variants are non-polymorphic in the target dataset. In these cases, variant-wise correlation r2 is mathematically undefined, because there is no variance in the observed genotypes. Our imputation benchmarks illustrate this problem clearly. For example, see Supplementary Note 5: in the [0.01%-0.1%) rpMAF category, setting undefined variant-wise correlation r2 values to zero resulted in an average r2 of 0.04 for GBR compared with 0.40 for YRI using the rpMAF-filtered UKB panel (or TOPMed r3 panel), despite the substantially closer ancestry match between GBR and the UK Biobank reference panel.

We also agree that pooled r2 can provide useful complementary information for very rare variants (including singletons) and therefore calculated this metric across samples and variants within each rpMAF category. While pooling avoids undefined variant-wise correlation r2 values, we observed that pooled correlation r2 remained high and showed comparatively little differentiation across the 26 subpopulations from the 1000 Genomes Project including populations with substantially different genetic similarity to the respective reference panels. We therefore consider pooled correlation r2 informative but also subject to limitations in these small, low-frequency benchmark datasets, because the pooled statistic is dominated by the large number of non-carrier observations and does not preserve variant-specific information.

We fully agree with the reviewer that MAE also has limitations for very rare variants, since the large number of correctly imputed major-allele genotypes can dominate the contribution of the relatively few minor-allele observations. We therefore retain MAE only as a complementary metric rather than as a replacement for correlation r2. In our imputation benchmarks, however, MAE remained more informative for distinguishing performance between populations and reference panels, including differences between well-matched populations such as GBR and less well-matched populations such as YRI.

We have revised Supplementary Section 5 accordingly to clarify the distinct limitations of variant-wise correlation r2, pooled correlation r2, and MAE for low-frequency variants in small target datasets.

The numbers refer to my points in the earlier review which the authors answer in their response.

1.-2. I am happy with the authors' response.

We are pleased that these points have been satisfactorily addressed.

3. The example the authors mention for YRI vs GBR in the rare variant bin shows that there are problems with the correlation r2 measure as it is being used by them, as they point out.

They seem to have put 0 for r2 when it cannot be computed. That obviously biases the mean towards 0. One plausible reason for the strange results they refer to is that, due to YRI being more polymorphic than GBR, there are fewer structural zeros in the r2 results for it.

The bottom line is that correlation r2 cannot be calculated for variants that are non-polymorphic in the target set. In that case, the pooled r2 result is much more reliable.

We agree with the reviewer. As correctly pointed out, the very low variant-wise correlation r2 values observed for GBR in the lowest-frequency bins result in part from the larger number of variants that are non-polymorphic in the target dataset and for which correlation r2 is therefore undefined. In our analyses, we set these undefined values to zero for the purpose of calculating mean variant-wise r2.

Assigning a default value of zero when an estimated imputation-quality R2 cannot be computed is also used as an implementation convention in some imputation software. However, we agree that applying a zero value to undefined empirical variant-wise correlation r2 inevitably biases the mean towards zero.

This explains, for example, why GBR can show lower average variant-wise r2 than YRI for very rare variants despite the substantially closer ancestry match between GBR and the UK Biobank reference panel.

We therefore agree that variant-wise correlation r2 is not appropriate for evaluating these non-polymorphic variants and that pooled correlation r2 provides a more suitable correlation-based measure in this setting. Accordingly, we additionally calculated pooled correlation r2 across all samples and variants within each rpMAF category.

Interestingly, in our 1000 Genomes Project benchmarks, pooled correlation r2 remained high across almost all 26 populations, including the lowest rpMAF category (0.0001 <= MAF < 0.001), with values >0.8 in nearly all cases, and showed comparatively little differentiation between worldwide populations that were well matched or less well matched to the UK Biobank reference panels. We therefore still present pooled r2 as an additional correlation-based assessment in the Supplementary Information, while retaining MAE in Fig. 2 of the main manuscript as a complementary metric that, in our benchmark, provides clearer differentiation between populations and the UK Biobank reference panel.

We have revised Supplementary Note 5 accordingly to clarify both the limitation of variant-wise r2 for non-polymorphic variants and the results obtained using pooled correlation r2.

4. The authors have misunderstood me regarding binning variants. Of course variants must be binned for summary statistics. By "to combine markers within a bin to estimate one r2 value", I was referring to the pooled r2 method.

I understand the reason for their MAF-based binning using the UKB reference panel since they are working with such small target sample sizes. That means though that many variants within a bin may be non-polymorphic in the target population. I agree that the TOPMed strategy of defining MAF bins is probably not applicable for UK Biobank, but that does not make the problem go away. They have their reason to define their bins. The problem with the correlation r2 is related to this approach.

I believe putting 0/1 for non-polymorphic variants to calculate statistics is not good. It can work when it is very rare (e.g., when the reference is very similar to the target data). I would select the pooled estimate instead here.

We agree that we had misunderstood the previous comment regarding combining variants within a bin and now understand that the reviewer was referring to the pooled correlation r2 approach. We also appreciate the reviewer's understanding of our rationale for defining the MAF bins based on the UK Biobank reference panel rather than on the target populations. As the reviewer points out, however, this approach inevitably results in a substantial number of variants within the low-frequency bins being non-polymorphic in some of the small target populations.

We therefore agree that assigning values of 0/1 to undefined variant-wise correlation r2 values is not an appropriate solution here and that pooled correlation r2 provides the more appropriate correlation-based measure. As described in our response to point 3, we still present pooled correlation r2 in the Supplementary Information. At the same time, we retain variant-wise r2 to illustrate its behavior in these small target datasets and MAE as a complementary metric, since in our benchmark data it provides additional differentiation between populations and reference panels. We have revised Supplementary Note 5 accordingly.

Finally, I would like to point out that inflating variant bins with non-polymorphic variants will bias statistics such as MAE to make the results look better.

We agree that the predominance of non-polymorphic variants can influence MAE values, particularly in the lowest rpMAF range, and should therefore be considered when interpreting differences across rpMAF categories.

However, this frequency-related effect does not compromise the relative comparison of imputation performance between reference panels within the same rpMAF category. Our use of MAE is focused primarily on relative performance differences between reference panels (UK Biobank versus TOPMed) and populations within the same rpMAF categories, rather than on absolute comparisons of MAE across different frequency categories.

We have therefore added the following sentence to the main text: "MAE values, particularly in the lowest rpMAF range category, also reflect the predominance of major-allele genotypes in small target datasets, which should be considered when interpreting absolute differences across rpMAF categories, whereas our analyses primarily focus on relative performance differences between reference panels within the same rpMAF category."

5. The sentence I referred to (599-601 in supplement) makes sense in relation to the example for YRI vs GBR in lines 586-590, but in between they refer to pooled correlation r2 results in Supplementary Tables 11-12 and Figures 19-22, which show a different scenario. I believe the sentence should come before referring to the pooled results. I for one went directly to the figures to see if the sentence made sense, since that was the last referred result before the sentence.

We thank the reviewer for notifying us about this potential misunderstanding. Our intention was to refer to the pooled correlation r2 results shown in Supplementary Figure 19-22 and Supplementary Tables 11-12. These results demonstrate that pooled correlation r2 values remained high across almost all target populations from the 1000 Genomes Project, including the lowest-frequency rpMAF category [0.01%-0.1%). The results further showed comparatively little differentiation between populations that were well or less well matching the UK Biobank or the TOPMed reference panels in terms of ancestry. We have revised the corresponding sentences in Supplementary Note 5.

6. I agree that the mean is much more used than the median, although I believe the median is more reliable. It was just an observation on my behalf. Using the same metric as the majority of the field does is not a problem.

The (very) minor comment has to do with the statement that knowing the mean gives indication of how many variants will pass a threshold (lines 91-93). Knowing the mean gives an indication of overall quality but only a weak indication of how many pass a threshold. If the mean happens to be very close to the threshold, you can expect around 50% to pass. If on the other hand it is far away, you only know that the percentage passing will be somewhat/quite lower/higher than 50%.

We agree with the reviewer and thank them for pointing this out. The average estimated R2 provides a useful summary of the overall imputation quality within an rpMAF category but does not provide a reliable estimate of the proportion of variants that will pass a particular R2 threshold. More generally, the proportion of variants exceeding a given threshold cannot be inferred from the mean alone and depends on the distribution of estimated R2 values within the category.

We have revised the corresponding sentence in the main manuscript to: ”The average estimated R2 for a given MAF category provides a summary of overall imputation quality within that category, while estimated R2 serves as a variant-wise quality criterion for post-imputation filtering”.

7.-8. I am happy with the authors' reply.

We are pleased that these points have been satisfactorily addressed.

I would like to finish by congratulating the authors on their good work and again emphasize that, even as it is, the paper merits publication.

We sincerely thank the reviewer for these very encouraging words and for the thoughtful and constructive feedback throughout the review process. We greatly appreciate the time and effort invested in reviewing and improving our manuscript.
